# 5-Methylcytosine depletion during tumour development: an extension of the miscoding concept.

**DOI:** 10.1038/bjc.1983.219

**Published:** 1983-10

**Authors:** J. Nyce, S. Weinhouse, P. N. Magee

## Abstract

We propose a general model for neoplastic development which postulates that the loss of methyl groups from 5-methylcytosines (5-mC) involved in the control of gene expression may initiate neoplastic transformation and give rise to the aberrant phenotype of the transformed cell. Interference with normal patterns of methylation can be envisioned to occur by a number of mechanisms: as a result of carcinogen-induced G:C leads to A:T transition leading to a loss of potentially methylatable cytosines; by mutations or chromosome rearrangement which disrupt the integrity of active DNA methylase genes; by separating methylated repressor regions of the genome from the genes they control; by direct interference with DNA methylation, as proposed for ethionine and 5-azacytidine; by spontaneous deamination of 5-mC to thymine, leading to accumulation of 5-mC:G leads to T:A transitions, by virus-induced perturbations in host cell methylation patterns; and by activation of DNA demethylases.


*Br. J. Cancer* (1983), **48**, 463–475

### Review

### 5-Methylcytosine depletion during tumour development: An extension of the miscoding concept

J. Nyce[1], S. Weinhouse[2] & P.N. Magee[3]

[1−3]*Fels Research Institute and* [3]*Departments of Pathology and* [2]*Biochemistry, Temple University School of Medicine, Philadelphia, Pa, 19140, and* [1]*Department of Biology, Temple University, Philadelphia, Pa, 19122, U.S.A.*

**Summary** We propose a general model for neoplastic development which postulates that the loss of methyl groups from 5-methylcytosines (5-mC) involved in the control of gene expression may initiate neoplastic transformation and give rise to the aberrant phenotype of the transformed cell. Interference with normal patterns of methylation can be envisioned to occur by a number of mechanisms: as a result of carcinogen-induced G⁝C→A:T transition leading to a loss of potentially methylatable cytosines; by mutations or chromosome rearrangement which disrupt the integrity of active DNA methylase genes; by separating methylated repressor regions of the genome from the genes they control; by direct interference with DNA methylation, as proposed for ethionine and 5-azacytidine; by spontaneous deamination of 5-mC to thymine, leading to accumulation of 5-mC⁝G→T:A transitions; by virus-induced perturbations in host cell methylation patterns; and by activation of DNA demethylases.

Perhaps the most ubiquitous aspect of tumours and the neoplastic cells of which they are composed is their loss of the ability to control cellular functions in a normal way. During the development of the malignant state, the neoplastic cell becomes increasingly refractory to both the internal and external stimulae which integrate its normal counterparts into functional tissues and organ systems. This lack of integration is associated with alterations in the expression of a host of gene products, including, for example, the ectopic biosynthesis of hormones (Rees, 1975; Imura, 1980), various nuclear modifications (Rovera, 1975; Sarma *et al.*, 1975), changes in enzyme and isozyme patterns (Weinhouse, 1970, 1980, Weinhouse *et al.*, 1972; Ibsen, 1977; Ghosh *et al.*, 1978; Foti *et al.*, 1977; Greengard & Herzfeld, 1977; Shapira *et al.*, 1963), appearance of foetal antigens (Gold, 1971) and cell surface modifications (Robin & Nicholson, 1975; Mora, 1974). It is thus clear that a powerful interference with the normal machinery of gene expression accompanies the transformed state. Such aberrant gene expression may yield to the transformed cell the required release from physiological control that defines neoplasia. Altered metabolism resulting from the appearance of isozymic forms may be the key both to such loss of host control and to the unbridled growth that are characteristic of rapidly growing tumours (Weinhouse, 1974).

As yet, altered gene expression has not been linked with the various processes known to produce tumours. Presented here is a model which proposes a basis for such a link, and which additionally provides a high degree of relevance to available data. The model is based upon a possible relationship between normal physiological methylation of DNA and chemically-induced alkylation of DNA, *viz.*, that carcinogen-induced alkylation leads to disappearance of sites of enzymatic methylation of DNA. Such hypomethylation would cause the expression of genes otherwise repressed, for example, those for isozymes, foetal antigens, ectopic hormones, etc.

### *Enzymatic methylation in gene expression*

There is increasing evidence that the enzymatic methylation of DNA at the 5-position of cytosine exerts some influence over the expression of eukaryotic genes, as suggested by Holliday & Pugh (1975), Riggs (1975) and Scarano (1971). McGhee & Ginder (1979) have shown that in adult reticulocytes and erythrocytes which are expressing or have expressed the adult β-globin gene, CCGG sites near the ends of the gene sequence are unmethylated, whereas in oviduct, brain and embryonic red blood cells, which do not express this gene, such sites are at least partially methylated. Christman *et al.* (1977) found that DNA isolated from Friend erythroleukaemic cells induced to produce globin mRNA is

Correspondence: J.W. Nyce, Fels Research Institute, Temple University School of Medicine, 3420 North Broad Street, Philadelphia, PA 19140.

Received 15 June 1983; accepted 14 July 1983.

© The Macmillan Press Ltd., 1983.

hypomethylated when compared to DNA from uninduced cells. Mandel & Chambon (1979) demonstrated the existence of a class of methylation sites ($m^{var}$) within and around the conalbumin, ovomucoid, and $\beta$-globin genes, the variable methylation of which correlated with transcriptional activity. When a gene region is in an active chromatin conformation, as measured by DNase I sensitivity (Kuo *et al.,* 1979), there is extensive hypomethylation at many of these $m^{var}$ sites. Sites of residual methylation near transcribed genes, as well as undermethylated sites near genes not being transcribed, were absent in DNase I-sensitive active chromatin. Based on these results, these authors suggest that in all tissues there is a certain possibility that an $m^{var}$ site is present in the unmethylated state; thus only undermethylation at a substantial number of $m^{var}$ sites of a given genomic region would be significantly correlated with the active state of chromatin. This and other information (for review see Doerfler, 1981) indicate that methylation at specific cytosine residues in DNA at specific times may be closely associated with the regulation of gene expression. Furthermore, the demonstration of specific unmethylated CG doublets in mammalian DNA by Browne *et al.* (1978) and the stringent conservation of the pattern of methylation throughout vertebrate evolution (Browne & Burdon, 1977) imply rigorous control of the DNA methylation system.

It has been suggested that 5-methylcytosine (5-mC) may provide signals for regulatory protein binding (Yuan & Meselson, 1970). Indeed, it is well established that the *Escherichia coli* K restriction and modification enzyme has a strong affinity for an unmethylated site but no detectable affinity for a methylated site. Sista *et al.* (1979) have shown that the 5-methyl group of thymine is the only important functional group recognized by the lac repressor at position 13 of the lac operator. Replacing thymine with cytosine resulted in a 7–8 fold decrease in lac repressor binding, whereas 5-mC bound repressor at least as tightly as the wild type sequence. Thus methylation is already known to affect the binding of sequence-specific proteins to prokaryotic DNA. It seems a reasonable working hypothesis that the insertion of a hydrophobic methyl group into the major groove of DNA, as occurs with the methylation of cytosine, might affect protein binding in eukaryotic systems as well.

### *Chemical alkylation of DNA*

Evidence has accumulated that non-enzymatic alkylation of DNA may also be biologically relevant. The site of alkylation of a nucleic acid, *in vivo* or *in vitro,* is greatly dependent upon the type of alkylating agent (Jensen, 1978; Singer *et al.,* 1978). In general, alkylating agents with low mutagenicity or carcinogenicity, such as dimethyl sulfate, are weak electrophiles and tend to attack primarily ring nitrogens. Agents that produce more reactive electrophilic species, such as N-nitroso and related compounds (Magee, 1977*a,* 1978), show an increasing ability to alkylate oxygen, reacting with all ring oxygens as well as phosphodiesters (Jensen & Reed, 1978; Singer, 1977, 1979). These compounds are very potent carcinogens, and many show an intriguing organ specificity (Druckrey, 1967).

Although N-nitroso and related compounds produce lesions at a variety of sites on DNA, alkylation at the $O^6$-position of guanine is thought to be responsible for the subsequent induction of tumours by these agents (Magee, 1976, 1979; Pegg & Nicoll, 1976; Lawley, 1980; Frei *et al.,* 1978). The persistence of $O^6$-alkylguanine in DNA of various rat and mouse tissues (Goth & Rajewsky, 1974; Kleihues & Margison, 1974; Margison *et al.,* 1976) correlates with the organ specificity of these compounds, especially when the rate of cell replication of the examined tissue is considered. The persistence of other lesions, such as those produced at the 7-position of guanine, the major site of alkylation, fails to predict which organs will serve as specific targets for tumorigenesis.

Goth & Rajewsky (1974) compared the persistence of $O^6$-ethylguanine in brain and liver of 10 day old rats treated with a single dose of N-ethyl-N-nitrosourea under conditions known to be selectively tumorigenic for brain but not liver. Molar fractions of $O^6$-ethylguanine, 7-ethylguanine, and 3-ethylguanine at one hour after injection were very similar in each tissue. The rate of removal of $O^6$-ethylguanine over a 240 h period, however, was much slower in brain than in liver. Nicoll *et al.* (1975) have shown that after a single large dose of dimethylnitrosamine, $O^6$-methylguanine is much longer lived in the kidney where tumours develop than in the liver, where they do not; and Kleihues & Margison (1974) found that in rats treated with methylnitrosourea, a potent neurogenic carcinogen which occasionally also produces tumours in the kidney but never in the liver, $O^6$-methylguanine is removed from DNA least rapidly in the brain, most rapidly in the liver, and at an intermediate rate in the kidney. The resistance of the liver and the susceptibility of the brain and kidney to tumorigenesis induced by a single dose of either ethylnitrosourea, dimethylnitrosamine or methylnitrosourea therefore appears to be related to the ability of these tissues to remove $O^6$-alkylguanine lesions from their respective DNAs.

**Figure 1** Diagrammatic representation of guanine alkylation-induced loss of methylatable cytosines following 2 or 3 rounds of replication. Original strands are shown in bold type, new strands in light type.

*Do the biological effects of enzymatic and chemical alkylation intersect?*

Based on the suggestion of Loveless (1969) that $O^6$-alkylation of guanine could lead to mispairing during DNA replication, Gerchman & Ludlum (1973) have shown in their *in vitro* transcription system that $O^6$-methylguanine mispairs with thymine in place of cytosine. Assuming that a similar mispairing event can occur *in vivo,* we suggest that the critical result of G:C→$O^6$-MeG:C→$O^6$-MeG:T→A:T transitions during DNA replication in N-nitrosamine-induced carcinogenesis is the loss of cytosine residues which may otherwise be potential sites of enzymatic methylation at the C-5 position (Figure 1). Such depletion of sites of enzymatic methylation may interfere with the otherwise rigidly fixed pattern of gene expression characteristic of fully differentiated cells. The aberrant gene expression and consequent unorchestrated attempt at differentiation that may result in a hypomethylated cell might then give rise, in both morphological and biological terms, to the transformed state. Transition mutations or other events (see below) leading to DNA hypomethylation are thus seen as capable of initiating the neoplastic phenotype by setting in motion programmes of transcription that disrupt the state of cellular differentiation.

*Evidence in support of the model*

Bryngelsson & Pero (1980) have presented evidence that the adenosine to guanine ratio of 1.272 in normal rat DNAs is significantly lower than that of 1.342 in tumours induced by a variety of agents, including 7,12-dimethylbenz(a)-anthracene, 3-methylcholanthrene, 3,4-benzo(a)pyrene, and 1,2-dimethylhydrazine. Their data indicate that ~1% of rat tumour DNA or one in every 50 base pairs has an altered purine or pyrimidine residue compared to normal rat DNAs. Kasten *et al.* (1982) found that, following DNA damage induced in human diploid fibroblasts by ultraviolet irradiation, N-methyl-N-nitrosourea, or N-acetoxy-2-acetylaminofluorene, repaired patches of DNA remained permanently undermethylated. According to Lapeyre & Becker (1979), rats treated with acetylaminofluorene, DNA from resulting premalignant hepatic nodules was undermethylated by 20%. Primary hepatocellular carcinomas found in these animals were undermethylated by 45% and diethylnitrosamine-induced hepatocellular carcinomas were undermethylated by 32.5%. Further evidence in favour of the model comes from work with the amino acid analogue and hepatic carcinogen, L-ethionine. Ethionine is only one carbon atom larger than the essential amino acid methionine, is metabolized via the same pathways as methionine (Stekol, 1963), and yet gives rise to numerous hepatocarcinomas when included in the diet (Farber, 1963). Although significant transfer of the ethyl group of ethionine to RNA occurs (Farber & Magee, 1960; Craddock, 1969), very little reaction with DNA is observed (Swann *et al.,* 1971; Ortwerth & Novelli, 1969). Significantly, Farber (1973) has pointed out that ethionine may be the exception to the rule that hepatocarcinogens damage DNA in a way detectable on alkaline sucrose gradients. It is impossible to rule out a role for direct ethylation of DNA in the mechanism of action of ethionine-induced carcinogenesis. However, levels of ethylation (Swann *et al.,* 1971; Ortwerth & Novelli,

1969; Grilli *et al.*, 1974), and of repair of induced DNA single strand breaks (Craddock & Henderson, 1978; Farber, 1973), approach insignificance when compared to levels induced by other hepatocarcinogens. Furthermore, only 7-ethylguanine (Swann *et al.*, 1971) and not $O^6$-ethylguanine or other miscoding and presumably oncogenic lesions, have been observed after ethionine treatment. This also contrasts with what is observed after treatment with other alkylating hepatocarcinogens, where $0^6$-alkylguanine occurs to a very significant extent (Magee *et al.*, 1976; Margison *et al.*, 1976; Nicoll *et al.*, 1977). We would like to offer, within the context of the model we are developing here, a possible mechanism of action for ethionine carcinogenesis which is an alternative to the direct DNA ethylation concept.

Smith & Salmon (1965) demonstrated that ethionine administration leads to the accumulation of S-adenosylethionine (SAE); and Cox & Irving (1977) found that SAE, as a metabolic analogue of S-adenosylmethionine (SAM), competitively inhibits DNA methylase *in vivo*, resulting in methyl-deficient DNA. Such inhibition of methylation by SAE is reversible upon replenishment of SAM pools, and the toxic and carcinogenic effects of ethionine can be reversed by the simultaneous administration of methionine (Farber & Ichinose, 1958; Brada *et al.*, 1976). Methionine may thus overcome the carcinogenic effects of ethionine by maintaining the ratio of SAM to SAE at a sufficiently high level to allow normal methylation of cytosine.

Since tRNA is ethylated by ethionine *in vivo*, it has been suggested that tRNA is the target for ethionine carcinogenesis (Borek & Kerr, 1971; Srinivasan & Borek, 1964). There are very little data to support this suggestion however, since there is increasing evidence that perturbation of DNA, and not RNA or other cellular macromolecules, initiates carcinogenesis (see below). Significantly, ethylated bases observed in tRNA of ethionine-treated animals have their methylated analogues in normal tRNA (Craddock *et al.*, 1968; Dunn, 1959, 1963; Smith & Dunn, 1959), suggesting that tRNA methylases operate with a relaxed specificity for alkyl group donor and can utilize either SAE or SAM. On the other hand, since 5-ethylcytosine is not found in DNA from ethionine-treated animals (Craddock, 1971), DNA methylases apparently possess a strict requirement for SAM. Because DNA methylases cannot utilize SAE, methylation of specific cytosines would be blocked in its presence. As this block continues throughout subsequent replications in the presence of concentrations of SAE capable of inhibiting DNA transmethylation reactions, the degree of cytosine hypomethylation would increase. The consequence would be an impaired gene regulation, leading to incomplete and abnormal differentiation. Since the replicative index of liver is very low, the model suggests that ethionine would be carcinogenic only at high concentration capable of inhibiting DNA methylase over extended periods. Such are the requirements for the induction of hepatic tumours by ethionine (Farber, 1963).

Other compounds that interfere with DNA methylation might also be expected to lead to changes in cellular differentiation and transformation. Whereas ethionine may inhibit DNA methylation by reducing cellular pools of SAM, 5-azacytidine (5-AzaCyt), an analogue of cytidine in which carbon 5 has been replaced by nitrogen, profoundly impairs methylation by masking the methyl acceptor site. Jones & Taylor (1980) have shown that 5-AzaCyt induces marked changes in differentiation of cultured mouse embryo cells. In testing other analogues substituted at the 5-position and elsewhere, these authors determined that changes in gene expression induced by such compounds correlated with their ability to inhibit DNA methylation.

Since certain DNA methylases ("maintenance methylases") may require hemimethylated double-stranded DNA as substrate (Pollack *et al.*, 1980; Bird, 1978), replications subsequent to a single treatment with 5-AzaCyt could produce fully unmethylated sequences, resulting in the permanent loss of 5-mC signal recognition sites for maintenance methylase activity. Constantinides *et al.* (1978) do, in fact, report that changes in the differentiated state induced in cell cultures by 5-AzaCyt occur by 8 to 11 cell divisions after treatment. At that time, the initial 5% substitution of 5-AzaCyt for cytosine bases in DNA have been reduced to a vanishingly small number. The 65% inhibition of DNA methylation observed by these authors in 5-AzaCyt-treated cultures would imply the erasing of previously established patterns of methylation by loss of a site of post-transcriptional modification. These observations suggest that 5-AzaCyt might be carcinogenic in tissues capable of incorporating it into their DNA. Likely targets might include rapidly replicating tissues such as the liver after partial hepatectomy, and the gastrointestinal tract. This suggestion may have some clinical relevance in view of the use of 5-AzaCyt as a chemotherapeutic agent (Armitage & Burns, 1977; Saiki *et al.*, 1978; Vogler *et al.*, 1976; Von Hoff *et al.*, 1976).

5-AzaCyt is mutagenic in *E. coli*, *Salmonella Typhimurium*, and V79 Chinese hamster cells (Fucik *et al.*, 1965; Marquardt & Marquardt, 1977), and limited evidence is available for its carcinogenic potential (Stoner *et al.*, 1973; NCI, 1978; IARC, 1981).

*Impaired methylation and hereditary tyrosinemia*

Methionine-deficient diets, which lead to decreased hepatic SAM pools, greatly potentiate the effects of a variety of liver carcinogens (Poirier *et al.*, 1977; Rogers & Newberne, 1980). These experimental results may have their natural counterpart in man in the disease known as hereditary tyrosinemia (HT). HT is a rare inborn metabolic disease characterized by hepatocellular and renal tubular dysfunction. Affected children usually die before the end of their second year, with hepatocarcinoma as the cause of death in most instances (Weinberg *et al.*, 1976). Notably, these individuals are unable to utilize methionine. Belanger *et al.* (1976) have shown that free hepatic methionine levels are severalfold higher than in control and are paralleled in the blood by high $\alpha$-fetoprotein (AFP) levels, a protein of unknown function usually found in significant amounts only in foetal liver, foetal serum and in many tumours. The high incidence of hepatoma in HT and the increased AFP-production with tumour growth suggest that the cells producing AFP are in a premalignant condition. Since AFP production correlates with the degree of methionine metabolism block in these patients, these authors have suggested that the normal ontogenic repression of AFP might depend on a methionine-related metabolic event, for example, the activation of a differentiation control mechanism working through a transmethylase pathway. This suggestion is based in part upon an observation by Gaull *et al.* (1970) that levels of methionine adenosyltransferase (MAT) are greatly decreased in hypermethionemic HT children. More recently Liau *et al.* (1979) reported that livers of children who died of HT showed abnormal MAT isozyme patterns. Such defects contrasted with normal development in which AFP repression and MAT expression are inversely related (Belanger *et al.*, 1976). Since hepatocytes of hypermethionemic HT patients have a depressed MAT activity, decreased SAM levels can be inferred and have indeed been reported (Forrester & Hancock, 1978). One can expect that reduced SAM pools would lead to hypomethylation of hepatic DNA in these children, just as it does in the animal models discussed above. We suggest that such hypomethylation of DNA may account for both the altered enzyme composition and the frequency of hepatocellular carcinoma observed in HT.

*Effects of pyridoxine deficiency*

In possibly related studies, Foy *et al.* (1974) found that diets deficient in pyridoxine, a required cofactor for transmethylation reactions, induced multiple atypical hyperplastic nodules in the livers of treated baboons and the appearance of AFP in serum. Such changes following pyridoxine deficiency were more severe than those observed following administration of the potent hepatocarcinogen, aflatoxin $B_1$. These workers further noted that histological changes in the livers of these baboons merely deprived of this specific dietary substance were the same as those observed in rats fed the hepatocarcinogen dimethylaminoazobenzene (DAB). Since both pyridoxine deficiency, by cofactor depletion, and DAB, possibly by mutagenic alteration of 5-mC sites, might induce a hypomethylated state in DNA, a unifying hypothesis to explain their similar effects can begin to be perceived. Because agents as diverse as ultraviolet radiation, ethionine, dimethylnitrosamine, dimethylhydrazine and acetylaminofluorene all have been shown to induce hypomethylation in their respective target tissues (Lapeyre & Becker, 1979; Cox and Irving, 1977; Kasten *et al.*, 1982; Nyce & Magee, unpublished observations), the hypothesis becomes potentially testable.

*Effects of choline deficiency*

A speculative but nonetheless cogent connection between possible hypomethylation and cancer is the long known and marked effects of choline and methionine deficiency in enhancing the incidence of liver cancer in rats fed such hepatocarcinogens as aflatoxin $B_1$, diethylnitrosamine, and N-2-fluorenylacetamide (Rogers & Newberne, 1980). A rôle for methionine follows directly from its role in transmethylation after conversion to S-adenosylmethionine. However, a molecular mechanism for a protective effect of choline on hepatocarcinogenesis has not been reported.

It has been known for many years that choline is oxidized by liver mitochondria to betaine and that betaine serves as a methyl donor to homocysteine in the formation of methionine (Meister, 1965; Skiba *et al.*, 1982).

$$1.\ (CH_3)_3\overset{+}{N}CH_2CH_2OH + 2\,0 \longrightarrow (CH_3)_3\overset{+}{N}CH_2COO^- + H_2O$$

$$2.\ (CH_3)_3\overset{+}{N}COO^- + HSCH_2CH_2CHNH_2COOH \longrightarrow CH_3SCH_2CH_2CHNH_2COOH + (CH_3)_2NCH_2COOH$$

Since choline deficiency exerts a carcinogenic action only in liver, (Rogers & Newberne, 1980) it is noteworthy that the enzyme, betaine-homocysteine methyltransferase, is active only in liver (Stekol, 1955; Meister, 1965). These conditions prompt the suggestion that the integrity of liver metabolism is dependent on methionine, and that choline is effective for this purpose in so far as it provides the methyl group for methionine synthesis in this organ.

*DNA repair, aging and viral carcinogenesis*

We have discussed O$^6$-alkylguanine as a significant lesion in tumour development because of its ability to eliminate methylation sites through G:C→A:T transitions. Because we are offering our model as a general description of the development of neoplasia, we wish to point out that any DNA adduct leading to the loss of methylation sites might be potentially tumorigenic. Conversely, genetic lesions which do not induce hypomethylation would not, according to the model, be carcinogenic.

Pfohl-Leskowitz *et al.* (1981) have shown that binding of 2-(acetylamino)-fluorene to C-8 of guanine inhibits DNA methylation irreversibly, thus possibly explaining the mechanism of carcinogenic action of this compound. In terms of carcinogen-DNA adducts leading to hypomethylation, one of the major predictions of our model is that alkylation of 5-mC moieties in DNA may be the most potently tumorigenic of all. Preliminary evidence from our laboratory suggests the formation of such lesions *in vitro*. Alkylation or other carcinogen-induced modification of 5-mC would significantly alter the character of the major groove of DNA. Any signal properties provided by 5-mC might consequently become impaired. Changes in the base pairing properties of 5-mC alkylation products are also possible. For example, alkylation at either O$^2$ or N$^4$ might give 5-mC a base pairing bond configuration more characteristic of thymine than cytosine. In replicating tissues, the resulting alkyl 5-mC:G→T:A transitions would create the same disturbance in gene expression and subsequent development of neoplasia postulated for O$^6$-alkylguanine-induced G:C→A:T transitions. Since 5-mC is not inserted into DNA *de novo* but occurs only by postreplicational methylation of cytosine, even the prompt repair of 5-mC alkylation products, if it occurs by a patch repair mechanism, may leave DNA in a hypomethylated state. With such repair, inserted cytosines must be remethylated prior to replication in order to prevent the heritable loss of hemimethylated substrate for maintenance methylase activity. One might guess, therefore, that a transmethylase similar to that observed for O$^6$-alkylguanine might be operative (discussed below). Repair effected by such a transmethylase would leave 5-mC in place, and hence be much less likely to upset the pattern of gene expression.

The model also offers an explanation for the observed increased susceptibility of older animals, including man, to spontaneous or chemically induced tumours (Ebbesen, 1974, 1977; Magee, 1978). Kudryashova & Vanyushin (1976*a*, *b*) and Vanyushin *et al.* (1980) have found that the DNA of old animals is hypomethylated when compared to that of young animals of the same sex and strain. That this may be due to spontaneous deamination of 5-mC to thymine is suggested by work with both prokaryotic and eukaryotic systems. Coulondre *et al.* (1978) have observed that spontaneous base substitution hotspots within the *lac* I gene of *E. coli* are due to deamination of 5-mC. Bird (1980) has discussed the relative scarcity of GC dinucleotides in vertebrate DNA along similar lines, providing evidence that 5-mC tends to mutate abnormally frequently to thymine. Although the deamination of adenosine to inosine and cytosine to uracil are also known to occur, the removal of these products can be effectively accomplished by the base excision repair system (Lindahl, 1979). However, since the deamination of 5-mC yields thymine, the excision of this product seems unlikely.

We suggest that spontaneous deamination of 5-mC to thymine, with the subsequent formation of 5-mC:G→T:A transitions (Figure 2), may explain the increased susceptibility to carcinogenesis that occurs with increasing age, since such transitions would accumulate over the lifetime of the animal. If relevant controlling regions become completely unmethylated as a result of such spontaneous deamination reactions, then the affected cell might

**Figure 2** Diagrammatic representation of gene activation by multiple 5mC:G→T:A transition mutations. Shading indicates inactive genes.

give rise to a "spontaneous" neoplasm. But even the partial methyl-depletion of multiply methylated controlling regions would increase the probability that a carcinogenic insult to DNA would result in a neoplastic effect. Simply stated, if "old" DNA is undermethylated, fewer hypomethylating mutations would be required to put it over the regulatory threshold.

A recent statistical analysis of tumours occuring in patients over 50 years old suggested that the tumours studied, and their numbers, could be related to a decrease in the integrity of the genome (Dix *et al.*, 1980). The spontaneous deamination of 5-mC and the subsequent generation and collection of 5-mC:G→T:A transitions may, according to our model, contribute to the age-associated decline in genome integrity suggested by these authors. If true, then chemical carcinogens might be said to exert their tumorigenic effects by mimicking a natural phenomenon.

Since the induction of tumours by viruses is well established, we wish to point out that variable patterns of DNA methylation in virus-transformed cells have been reported (Rubery & Newton, 1973; Browne & Burdon, 1977; Greene *et al.*, 1975; DeWichter *et al.*, 1971; Berneman *et al.*, 1978). Groudine *et al.* (1981) recently showed that the 5-AzaCyt-induced hypomethylation of an endogenous retroviral gene of chick embryo erythrocytes results in the activation of this genome as judged by DNAse sensitivity, transcription and synthesis of viral proteins. Other observations relating DNA hypomethylation to viral carcinogenesis have been made. For example, proviral mouse mammary tumour virus (MMTV) DNA sequences acquired from milk in animals at high risk for breast cancer are undermethylated compared to endogenous MMTV sequences that are associated with a much reduced risk (Breznik & Cohen, 1982). Since an inverse relationship has been demonstrated between proviral methylation and transcriptional activity, derepression of endogenous proviruses through demethylation of these sequences may represent the mechanism of MMTV-induced tumorigenesis (Breznik & Cohen, 1982). Analysis of the methylation pattern of the viral thymidine kinase (TK) gene in Herpes simplex virus (HSV)-transformed mouse cells showed that when the gene was being actively transcribed it was unmethylated, when inactive it was methylated, and when induced to activity by 5-AzaCyt it was again unmethylated. This finding was extended by Waechter & Baserga (1982) who found that when the cloned gene for HSV-TK was methylated with Eco Rl methylase and microinjected into the nucleus of $TK^{(-)}$ cells, methylation at particular sites markedly reduced or abolished the expression of the gene. These authors pointed out the possibility that different genes might respond differently to methylation, since similar Eco Rl methylation of the gene coding for Simian virus 40 T antigen had no effect upon its expression after microinjection. A causative relationship between DNA methylation and decreased gene expression in Herpes simplex TK genes was also observed by Christy & Scangos (1982). Diala & Hoffman (1982) observed hypomethylation of HeLa cell DNA and the absence of 5-mC in SV40 and adenovirus (Type 2) DNA. Desrosiers *et al.* (1979) observed that viral DNA in cells of *Herpesvirus saimiri* transformed non-producing lymphoid cell lines contains DNA methylated at cytosine positions which are unmethylated in virion DNA and in DNA of lymphoid cell lines that produce virus. In other studies an inverse correlation was observed between the levels of methylation of integrated adenovirus (Type 12) DNA sequences and viral gene expression in transformed hamster cells (Sutter & Doerfler, 1980). These results suggest that hypomethylation of DNA may provide a common transforming mechanism shared by chemical carcinogens and oncogenic viruses.

*Genetic* vs. *epigenetic considerations*

A purely epigenetic model of carcinogenesis, also concerned with the effects of hypomethylation upon cell populations, has been put forward (Holliday, 1979). This model proposes that damage to DNA results in the potentially reversible loss of methyl groups as a function of repair processes. This would occur either as a result of repair before DNA synthesis, and before appropriate methylases have remethylated newly inserted cytosines, or as a result of recombination following damage to DNA.

There are, however, observations which detract from this otherwise attractive hypothesis. Thus, while the persistence of $O^6$-alkylguanine correlates well with the tumour susceptibility of various tissues and that of 7-alkylguanine does not, these lesions would be predicted by the purely epigenetic model to be equally tumorigenic if sequences containing them were excised, then repaired, but not remethylated prior to DNA replication. Yet methyl methanesulfonate (MMS), which produces extensive methylation at the $N_7$ position of guanine and only insignificant amounts of the $O^6$-alkylated product, is not hepatocarcinogenic in the rat even when administered in the wave of DNA replication which follows partial hepatectomy (Craddock, 1975). Furthermore, evidence has accumulated which suggests that, while 7-alkylguanine is removed from DNA by a base excision process presumably susceptible to repair-associated DNA hypomethylation, $O^6$-alkylguanine is repaired in a transmethylase reaction where base integrity remains intact (Olson & Lindahl, 1980; Pegg *et al.*, 1982; Waldstein *et al.*, 1982; Renard *et al.*, 1981; Regan & Setlow, 1974). Thus the repair mechanism for $O^6$-alkylguanine does not proceed by an excision system that would deplete 5-mC. Sequences containing methylated cytosines in the vicinity of such lesions remain intact. Since there are no gaps to fill, there is no reason to expect that $O^6$-alkylguanine repair would lead to hypomethylation. The repair-associated hypomethylation model therefore suffers from the flaw that it predicts that 7-alkylguanine, but not $O^6$-alkylguanine, is a critical oncogenic lesion, when exactly the opposite seems to be true. It would appear, then, that fixation of $O^6$-alkylguanine lesions via G:C→A:T transition mutations is a more likely mechanism of carcinogenesis, at least for alkylating carcinogens, than that postulated in the purely epigenetic model.

The molecular basis underlying the relationship between mutagenesis and carcinogenesis has become increasingly substantial (see Magee, 1977*b*). Thus, most chemical carcinogens are also mutagens (Ames & McCann, 1976; McCann & Ames, 1977); tumours appear to be clonal in origin, suggesting the occurrence within a single cell of a permanent molecular alteration (Gould *et al.*, 1978); cells that are more sensitive to mutagenic lesions are also more sensitive to malignant transformation (Cleaver & Bootsma, 1975; Mortelman *et al.*, 1976; Takebe *et al.*, 1977); mutagenic metabolites produced by metabolic activation within tumour-susceptible organs have been identified as ultimate carcinogens (Felton & Nebert, 1975); and the direct perturbation of DNA has been shown to be sufficient to initiate neoplastic transformation (Barrett *et al.*, 1978). Yet Cairns (1981) has convincingly championed the argument that many cancers may be caused by non-mutational mechanisms (e.g., cancers arising in tissues next to implanted sheets of plastic, or induced by implantation of ovary or embryo cells into sites where constraints upon their cellular multiplication are removed). He has concluded that there is no obvious connection between the many ways that tumours can be induced. One strength of the hypomethylation model is that it provides such a common link between diverse carcinogenic stimulae. Another strength is that it provides a basis for believing the difference between mutational and epigenetic mechanisms may be more apparent than real, with both mechanisms sharing considerable overlap. As an example, one may consider the loss of hemimethylated sites for DNA methylase that would occur if 5-mC were to be modified by an alkylating carcinogen, repaired but not remethylated before the next round of DNA synthesis. Similarly, interference with DNA methylase induced by ethionine could lead to permanent alteration in the pattern of methylation of the genetic material. Are such changes in the expressible nature of the involved DNA to be considered mutational or not? They do, after all, disrupt the clonal inheritance of the genomic methylation pattern (Riggs, 1975; Holliday & Pugh, 1975). Yet, in the case of ethionine-induced hypomethylation, if a sequence-specific methylase capable of acting upon completely unmethylated sites could be induced, the methylation pattern could theoretically be fully restored. In this scenario, the boundaries between epigenetic and mutational mechanisms of carcinogenesis become obscure.

We might point out here that short-term bacterial mutagenesis assays may produce some false negative results when testing the relationship between carcinogenesis and mutagenesis because of differences in the methylated bases occurring in prokaryotes and eukaryotes, and their different functions. For example, a compound like ethionine which is not overtly genotoxic does not test positively in a *Salmonella typhimurium* revertant assay (McCann *et al.*, 1975), yet is clearly carcinogenic in eukaryotes. The number of other substances tested which are either mutagens or carcinogens but not both offer perhaps one of the most intriguing avenues for research into the basic mechanism of tumorigenesis.

### *Cellular oncogenes*

While the model presented here implies widespread changes in gene expression as a result of chemically-induced hypomethylation of DNA, it is entirely possible that only one or a few of the newly activated genes are responsible for transformation. Much of the recent work on cellular oncogenes suggests that this may be the case (for a review see Weinberg, 1982). From our point of view, the cellular oncogene (or oncogenes) would be one or a few of many genes activated during carcinogen-induced hypomethylation. Certain retro-viruses incorporate into their DNA a cellular gene which is thus released from host control (Weinberg, 1981). This collapse of host regulatory function for the oncogene might involve actual demethylation of the host sequence incorporated into the virion (Breznik & Cogen, 1982) or physical separation of the oncogene from the methylated host DNA sequence responsible for its repression. Integration of the avian leukosis viral genome next to a specific cellular oncogene has been shown to occur, and to transcriptionally activate the gene (Hayward *et al.*, 1981).

Possible candidates for genes with oncogenic potential may include those genes for 5-mC demethylase or 5-mC deaminase. While the former activity has been reported in murine erythroleukemic cells (Gjerset & Martin, 1982), no evidence exists for the latter, although its function during early development remains an attractive theoretical possibility (Holliday & Pugh, 1975). Since the levels of hypomethylation following treatment with alkylating agent appear to be larger than would be expected based upon the number of pro-mutagenic $O^6$-alkylguanine lesions induced, it is possible that low level hypomethylation occasionally releases the gene for a putative 5-mC demethylase from repression, initiating a cascade of newly expressed genes which release the cell from growth control.

*Tumour promotion*

An early and profound effect of tumour promoters is the stimulation of mitotic activity and cell proliferation (Diamond *et al.*, 1980). All that may be required of a promoter is that it induce a condition of chronic regenerative hyperplasia in the target tissue (Argyris, 1981). Since replication is required in order for hypomethylation-inducing lesions to become fixed in DNA, promoters may exert their effect primarily by increasing the rapidity of cell turnover. As can be seen in Figure 1, an initiating event such as the formation of $O^6$-alkylguanine or spontaneous deamination of 5-mC to thymine will not be expressed until successive DNA replications lead to hypomethylation.

This would be consistent with the long latent periods observed between initiation and administration of promoter that are possible in some experimental systems. Once the genetic damage has been permanently fixed into the DNA in the form of lost sites of enzymatic methylation, many months or even years may elapse before that cell is induced to undergo proliferation, or does so spontaneously. When proliferation is resumed, however, the undermethylated genome may allow the expression of previously repressed genes, some of which may release the cell from growth control. We conclude our discussion by drawing attention to Table I. It is remarkable that in every system in which DNA hypomethylation is encountered, neoplasia follows. It is, of course, possible that this accumulation of circumstantial evidence may be misleading, and it is always difficult to separate cause from effect, especially in a subject as complex as neoplastic transformation. Nevertheless, the correlation between DNA hypomethylation and neoplastic transformation is intriguing and certainly deserving of serious investigation.

**Table I** Systems in which hypomethylation of DNA may initiate the transformed state

| AGENT OR CONDITION AFFECTING DNA METHYLATION | AFFECTED SYSTEM | MECHANISM OF INDUCTION OF DNA HYPOMETHYLATION |
|---|---|---|
| Chemical alkylation (e.g., by N—nitroso—or related compounds) | DNA | G:C → A:T transition mutations which delete present or potential sites of enzymatic DNA methylation |
| Other carcinogen—induced modifications (e.g. Benzo (a) Pyrene, aflatoxin B1, AAF, DAB) | DNA | E.g., AAF insertion—denaturation of DNA and subsequent irreversible inhibition of DNA methylase (Pfohl—Leskowicz et al, 1981) |
| Ethionine, hereditary tyrosinemia, choline/methionine deficiency | DNA methylase system | Reduction of SAM pool size and subsequent inhibition of DNA methylase |
| Pyridoxine deficiency | DNA methylase system | Cofactor depletion and subsequent inhibition of DNA methylase |
| 5—azacytidine | DNA | Loss of methylatable cytosine residues in CpG sequences |
| Aging | DNA | 5mC:G → T:A transition mutations due to spontaneous deamination |
| Viruses | Host DNA, DNA methylase system | a) differential methylation of host vs viral DNA by virus associated DNA methylase? b) insertion mutation? |

### References

AMES, B.N. & MCCANN, J. (1976). Carcinogens are mutagens: A simple test system. In *Screening Tests in Chemical Carcinogenesis*. (Eds. Montessano *et al*.). International Agency for Research on Cancer: Lyon, Vol. **12**, p. 493.

ARGYRIS, T.S. (1982). Tumour promotion by regenerative epidermal hyperplasia in mouse skin. *J. Cutaneous Pathol.*, **9**, 1.

ARMITAGE, J.O. & BURNS, C.P. (1977). Treatment of refractory adult acute nonlymphoblastic leukemia with subcutaneous 5-Azacytidine. *Cancer Treat. Rep.*, **61**, 1721.

BARRETT, J.C., TSUTSUI, T. & TS'O, P.O.P. (1978). Neoplastic transformation induced by a direct perturbation of DNA. *Nature*, **274**, 229.

BELANGER, L., LAROCHELLE, J., BELANGER, M. & PRIVE, L. (1976). Tyrosinosis: Hereditary persistence of alpha-1-fetoprotein. In *Onco-Developmental Gene Expression*. (Eds. Fishman & Sell). Academic Press: New York, p. 155.

BERNEMAN, A., ROBERT-GERO, M. & VIGIER, P. (1978). DNA methylase activity associated with Rous sarcoma virus. *FEBS Letters*, **89**, 33.

BIRD, A.P. (1978). Use of restriction enzymes to study eukaryotic DNA methylation: I. The methylation pattern in ribosomal DNA from *Xenopus laevis*. *J. Mol. Biol.*, **118**, 49.

BIRD, A.P. (1980). DNA methylation and the frequency of CpG in animal DNA. *Nucl Acids Res.*, **8**, 1499.

BOREK, E. & KERR, S.J. (1972). Atypical transfer RNAs and their origin in neoplastic cells. *Adv. Cancer Res.*, **15**, 163.

BRADA, Z., BULBA, S. & ALTMAN, N.H. (1976). The influence of DL-methionine on the metabolism of S-adenosylethionine in rats chronically treated with DL-ethionine. *Cancer Res.*, **36**, 1973.

BREZNIK, T. & COHEN, J.C. (1982). Altered methylation of endogenous viral promoter sequences during mammary carcinogenesis. *Nature*, **295**, 255.

BROWNE, M.J. & BURDON, R.H. (1977). The sequence specificity of vertebrate DNA methylation. *Nucl Acids Res.*, **4**, 1025.

BROWNE, M.J., CATO, A.C.B. & BURDON, R.H. (1978). The distribution of modified and non-modified C-G doublets in BHK-21 cell DNA. *FEBS Letters*, **91**, 69.

BRYNGELSSON, T. & PERO, R.W. (1980). Purine base composition analysis of normal and tumor rat DNA. *Chem. Biol. Interact.*, **29**, 267.

CAIRNS, J. (1981). The origin of human cancers. *Nature*, **289**, 353.

CHRISTMAN, J.K., PRICE, P., PEDRINAN, L. & ACS, G. (1977). Correlation between hypomethylation of DNA and expression of globin genes in Friend erythroleukemic cells. *Eur. J. Biochem.*, **81**, 53.

CHRISTY, B. & SCANGOS, G. (1982). Expression of transferred thymidine kinase genes is controlled by methylation *Proc. Natl Acad. Sci.*, **79**, 6299.

CLEAVER, J.E. & BOOTSMA, D. (1975). Xeroderma pigmentosum: Biochemical and genetic characteristics In *Annual Rev. Genetics*. (Ed. Roman) Annual Reviews, Inc: Palo Alto, p. 19.

CONSTANTINIDES, P.G., TAYLOR, S.M. & JONES, P.A. (1978). Phenotypic conversion of cultured mouse embryo cells by azapyrimidine nucleosides. *Dev. Biol.*, **66**, 57.

COULONDRE, C., MILLER, J.H., FARBAOUGH, P.J. & GILBERT, W. (1978). Molecular basis of base substitution hotspots In *Escherichia coli*. *Nature*, **274**, 775.

COX, R. & IRVING, C.C. (1977). Inhibition of DNA methylation by S-adenosylethionine with the production of methy-deficient DNA in regenerating rat liver. *Cancer Res.*, **37**, 222.

CRADDOCK, V.M. (1969). Methylation of transfer RNA and of ribosomal RNA in rat liver in the intact animal and the effect of carcinogens. *Biochem. Biophys. Acta.*, **195**, 351.

CRADDOCK, V.M. (1971). Methylation of DNA in the intact animal and the effect of the carcinogens dimethylnitrosamine and ethionine. *Biochem. Biophys. Acta.*, **240**, 376.

CRADDOCK, V. M. (1975). Effect of a single treatment with the alkylating carcinogens dimethylnitrosamine, diethylnitrosamine and methylmethane-sulphonate on liver regenerating after partial hepatectomy. *Chem. Biol. Interact.*, **10**, 313.

CRADDOCK, V.M. & HENDERSON, A. R. (1978). *De novo* and repair replication of DNA in liver of carcinogen-treated animals. *Cancer Res.*, **38**, 2135.

CRADDOCK, V.M., VILLA-TREVENO, S. & MAGEE, P.N. (1968). Occurrence of 7-methylguanine in nucleic acids of rat liver. *Biochem. J.*, **107**, 179.

DESROSIERS, R.C., MULDER, C. & FLECKENSTEIN, B. (1978). Methylation of herpes virus saimuri DNA in lymphoid tumor cell lines. *Proc. Natl Acad. Sci.*, **76**, 3839.

DEWICHTER, R., MERREGAERT, J., VANDENBERGHE, A., CONTRERAS, R. & FIERS, W. (1971). Studies on the bacteriophage MS2. The untranslated 5'-terminal nucleotide sequence preceding the first cistron. *Eur. J. Biochem.*, **22**, 400.

DIALA, E.S. & HOFFMAN, R.M. (1982). Hypomethylation of HeLa cell DNA and the absence of 5-methylcytosine in SV40 and adenovirus (Type 2) DNA: Analysis by HPLC. *Biochem. Biophys. Res. Commun.*, **107**, 19.

DIAMOND, L., O'BRIEN, T.G. & BAIRD, W.M. (1980). Tumor promoters and the mechanism of tumor promotion. *Adv. Cancer Res.*, **32**, 20.

DIX, D., COHEN, P. & FLANNERY, A. (1980). On the role of aging in cancer incidence. *J. Theor. Biol.*, **83**, 163.

DOERFLER, W.J. (1981). DNA methylation—A regulatory signal in eukaryotic gene expression. *Gen. Virol.*, **57**, 1.

DRUCKREY, H. (1967). Organotropic carcinogenic effects of 65 different N-nitroso compounds in BD rats. *Z. Z. Krebsforsch.*, **69**, 103.

DUNN, D.B. (1959). Additional components in RNA of rat liver fractions. *Biochem. Biophys. Acta.*, **34**, 286.

DUNN, D.B. (1963). The isolation of 1-methyladenylic acid and 7-methylguanylic acid from RNA. *Biochem. J.*, **86**, 14.

EBBESEN, P. (1974). Aging increases susceptibility of mouse skin to DMBA carcinogenesis. *Science*, **183**, 217.

EBBESEN, P. (1977). Effect of age of non-skin tissues on susceptibility of skin grafts to 7,12-dimethylbenz(a)-anthracene (DMBA) carcinogenesis in BALB/c mice, and effect of age of skin graft on susceptibility of surrounding recipient skin to DMBA. *J. Natl Cancer Inst.*, **58**, 1057.

FARBER, E. (1963). Ethionine carcinogenesis. *Adv. Cancer Res.*, **7**, 380.

FARBER, E. (1973). Carcinogenesis–Cellular evolution as a unifying thread. *Cancer Res.*, **33**, 2537.

FARBER, E. & ICHINOSE, H. (1958). The prevention of ethionine-induced carcinoma of the liver in rats by methionine. *Cancer Res.*, **18**, 1209.

FARBER, E. & MAGEE, P.N. (1960). The probable alkylation of liver RNA by the hepatic carcinogens dimethylnitrosamine and ethionine. *Biochem. J.* **76**, 58.

FELTON, J.S. & NEBERT, D.W. (1975). Mutagenesis of certain activated carcinogens *in vitro* associated with genetically mediated increases in monooxygenase activity and cytochrome P-450. *J. Biol. Chem.*, **250**, 6769.

FORRESTER, P.I. & HANCOCK, R.L. (1978). Theoretical mechanisms for synthesis of carcinogen-induced embryonic proteins. I. Alpha-fetoprotein induction by ethionine. *Med. Hypothesis*, **4**, 31.

FOTI, A.G., KERSCHMAN, H. & COOPER, J.F. (1977). Isozymes of acid phosphatase in normal and cancerous prostatic tissue. *Cancer Res.*, **37**, 4120.

FOY, H., KONDI, A., DAVIDES, J.N.P. & 4 others. (1974). Histologic changes in livers of pyridoxine-deprived baboons; relation to alpha-1-fetoprotein and liver cancer in Africa. *J. Natl Cancer Inst.*, **53**, 1295.

FREI, J.V., SWENSEN, D.H., WARREN, W.W. & LAWLEY, P.D. (1978). Alkylation of DNA *in vivo* in various organs of C57BL mice by the carcinogens N-methyl-N-nitrosourea, N-ethyl-N-nitrosourea and ethyl-methanesulfonate in relation to the induction of thymic lymphoma: some applications of high pressure liquid chromatography. *Biochem.*, **174**, 1031.

FUCIK, V., ZANDRAEL, S., SORMOVA, Z. & SORM, F. (1965). Mutagenic effect of 5-azacytidine in bacteria. *Coll. Czechov. Chem. Comm.*, **30**, 2883.

GAULL, G.E., RASSIN, D.K., SOLOMON, G.E., HARRIS, R.C. & STURMAN, J.A. (1970). Biochemical observations on so-called hereditary tyrosinemia. *Pediatr. Res.*, **4**, 337.

GERCHMAN, L.L. & LUDLUM, D.B. (1973). The properties of $0^6$-methylguanine in templates for RNA polymerase. *Biochem. Biophys. Acta.*, **308**, 310.

GHOSH, B.C., GHOSH, L., NEWSON, B.L. & DAS GUPTA, T.K. (1978). Histochemical and ultrastructural study of lactic dehydrogenase in chemically induced lung cancer. *Cancer Res.*, **38**, 2790.

GJERSET, R.A. & MARTIN, Jr., D.W. (1982). Presence of DNA demethylation activity in the nucleus of murine erythroleukemic cells. *J. Biol. Chem.*, **257**, 5881.

GOLD, P. (1971). Embryonic origin of human tumor-specific antigens. *Prog. Exp. Tumor Res.*, **14**, 43.

GOTH, R. & RAJEWSKY, M.F. (1974). Molecular and cellular mechanisms associated with pulse-carcinogenesis in the rat nervous system by ethylnitro-sourea: ethylation of nucleic acids and elimination rates of ethylated bases from the DNA of different tissues. *Z. Krebsforsch*, **82**, 37.

GOULD, M.N., JIRTLE, CROWLE, J. & CLIFTON, K.H. (1978). Reevaluation of the number of cells involved in the neutron induction of mammary neoplasms. *Cancer Res.*, **38**, 189.

GREENE, P.H., POONIAN, M.S., NUSSBAUM, A.L. & 4 others. (1975). Restriction and modification of a self-complementary octanucleotide containing the Eco R1 substrate. *J. Med. Biol.*, **99**, 237.

GREENGARD, O. HERZFELD, A. (1977). The undifferentiated enzymic composition of human fetal lung and pulmonary tumors. *Cancer Res.*, **37**, 884.

GRILLI, S., FERRERI, A.M., ROCCHI, P. & PRODI, G. (1974). *In vivo* and *in vitro* binding of ethionine with nucleic acids. *Gann.*, **65**, 507.

GROUDINE, M., EISENMAN, R. & WEINTRAUB, H. (1981). Chromatin structure of endogenous retroviral genes and activation by an inhibitor of DNA methylation. *Nature*, **292**, 311.

HAYWARD, W.S., NEEL, B.G. & ASTRIN, S.M. (1981). Activation of a cellular onc gene by promotor insertion in ALU-induced lymphoid leukosis. *Nature*, **290**, 475.

HOLLIDAY, R. (1979). A new theory of carcinogenesis. *Br. J. Cancer*, **40**, 513.

HOLLIDAY, R. & PUGH, J.E. (1975). DNA modification mechanisms and gene activity during development. *Science*, **187**, 226.

IBSEN, K.H.(1977). Interrelationships and functions of the pyruvate kinase isozymes and their variant forms. *Cancer Res.*, **37**, 341.

IMURA, H. (1980). Ectopic hormone production viewed as an abnormality in regulation of gene expression. *Adv. Cancer Res.*, **33**, 39.

INTERNATIONAL AGENCY FOR RESEARCH ON CANCER. (1981). Some antineoplastic and immuno-suppressive agents. In *IARC Monographs on the Evaluation of the Carcinogenic Risk of Chemicals to Humans*, Lyon, Vol. **26**, p. 37.

JENSEN, D.E. (1978). Reaction of DNA with alkylating agents. Differential alkylation of poly[dA-dT] by methylnitrosourea and ethylnitrosourea. *Biochemistry*, **17**, 5108.

JENSEN, D.E. & REED, D.J. (1978). Reaction of DNA with alkylating agents. Quantitation of alkylation by ethyl-nitrosourea of oxygen and nitrogen sites on poly[dA-dT] including phosphotriester formation. *Biochemistry*, **17**, 5098.

JONES, P.A. & TAYLOR, S.M. (1980). Cellular differentiation, cytidine analogs and DNA methylation. *Cell*, **20**, 85.

KASTEN, M.B., GOWANS, B.J. & LIEBERMAN, M.N. (1982). Methylation of deoxycytidine incorporated by excision-repair synthesis of DNA. *Cell*, **30**, 509.

KLEIHUES, P. & MARGISON, G.P. (1974). Carcinogenicity of N-methy-N-nitrosourea: possible role of excision repair of $0^6$-methylguanine from DNA. *J. Natl Cancer Inst.*, **53**, 1839.

KUDRYASHOVA, I.B. & VANYUSHIN, B.F. (1976*a*). Methylation of rat liver nuclear DNA during induction by hydrocortisone. *Biokhimiya*, **41**, 215.

KUDRYASHOVA, I.B. & VANYUSHIN, B.F. (1976*b*). Methylation of nuclear DNA from different rat organs *in vitro*: Tissue and age differences in acceptor capacity. *Biokhimiya*, **41**, 1106.

KUO, M.T., MANDEL, J.L. & CHAMBON, P. (1979). DNA methylation: correlation with DNAse I sensitivity of chicken ovalbumin and conallumin chromatin. *Nucleic Acids Res.*, **7**, 2105.

LAPEYRE, J.N. & BECKER, F.F. (1979). 5-Methylcytosine content of nuclear DNA during chemical hepatocarcinogenesis and in carcinomas which result. *Biochem. Biophys. Res. Commun.*, **87**, 698.

LAWLEY, P.D. (1980). DNA as a target of alkylating carcinogens. *Br. Med. Bull.*, **36**, 19.

LIAU, M.C., CHANG, C.F., BELANGER, L. & GRENIER, A. (1979). Correlation of isozyme patterns of S-adenosylmethionine synthetase with fetal stages and pathological states of the liver. *Cancer Res.*, **39**, 162.

LINDAHL, T. (1979). DNA glycosylases, endonucleases for apurinic/apyrimidinic sites, and base excision repair. *Prog. Nucleic Acid Res. Mol. Biol.*, **22**, 135.

LOVELESS, A. (1969). Possible relevance of $0^6$ alkylation of deoxyguanosine to mutagenicity of nitrosamines and nitrosamides. *Nature*, **223**, 206.

MAGEE, P.N. (1976). The role of the liver in chemical carcinogenesis. *Panminerva Med.*, **18**, 427.

MAGEE, P.N. (1977*a*). Evidence for the formation of electrophilic metabolites from N-nitroso compounds. In: *Origins of Human Cancer*, Cold Spring Harbor Laboratory, p. 629.

MAGEE, P.N. (1977*b*). The relationship between mutagenesis, carcinogenesis and teratogenesis In: *Progress in Genetic Toxicology*. (Eds Scott *et al.*), Elsevier/North-Holland Biomedical Press: Amsterdam, p.15.

MAGEE, P.N. (1978). Carcinogenesis and aging. In: *Pharmacological Intervention in the Aging Process*. (Eds. Robert *et al.*), Plenum: New York, p. 133.

MAGEE, P.N. (1979). Organ specificity of chemical carcinogens. *Adv. Med. Oncol. Res. Ed.*, **1**, 213.

MAGEE, P.N., MONTESANO, R. & PREUSSMANN, R. (1976). N-Nitroso compounds and related carcinogens. *ACS Monogr.*, **173**, 491.

MANDEL, J.L. & CHAMBON, P. (1979). DNA methylation: organ specific variations in the methylation pattern within and around avalbumin and other chicken genes. *Nucleic Acids Res.*, **7**, 2081.

MARGISON, G.P., MARGISON, J.M. & MONTESANO, R. (1976). Methylated purines in the DNA of various Syrian-golden-hamsters tissues after administration of a hepatocarcinogenic of dimethylnitrosamine. *Biochem. J.*, **157**, 627.

MARQUARDT, H. & MARQUARDT, H. (1977). Induction of malignant transformation and mutagenesis in cell cultures by cancer chemotherapeutic agents. *Cancer*, **40**, 1930.

MCCANN, J. & AMES, B.N. (1977). The *Salmonella* microsome mutagenicity test: Predictive value for animal carcinogenicity. In *Origins of Human Cancer*. (Eds. Hiatt *et al.*) Cold Spring Harbor Laboratory: p. 1431.

MCCANN, J., CHOI, E., YAMASAKI, E. & AMES, B.N. (1975). Detection of carcinogens as mutagens in the *Salmonella*/microsome test: Assay of 300 chemicals. *Proc. Natl Acad. Sci.*, **71**, 5135.

MCGHEE, J.D. & GINDER, G.D. (1979). Specific DNA methylation sites in the vicinity of the chicken $\beta$-globin genes. *Nature*, **280**, 419.

MEISTER, A. (1965). *Biochemistry of the Amino Acids, Vol.* **2.** Academic Press: New York, p. 757.

MORA, P.T. (1974). *Cell Surfaces and Malignancy*. DHEW Publication No. NIH 75, 796.

MORTELMAN, K., FRIEDBERG, E.C., SLOR, H., THOMAS, G. & CLEAVER, J.E. (1976). Defective-thymine dimer excision by cell-free extracts of xenoderma pigmentosum cells. *Proc. Natl Acad. Sci.*, **73**, 2757.

NATIONAL CANCER INSTITUTE TECHNICAL REPORT SERIES NO. 42. (1978). DHEW Publ. No. (NIH) *Bioassay of 5-Azacytidine for Possible Carcingenicity* 78–842 Washington, D.C., U.S. Gov't Printing Office.

NICOLL, J.W., SWANN, P.F. & PEGG, A.E. (1975). Effect of dimethylnitrosamine on the persistence of methylated guanines in rat liver DNA. *Nature*, **254**, 261.

NICOLL, J.W., SWANN, P.F. & PEGG, A.E. (1977). The accumulation of $0^6$-methylguanine in the liver and kidney DNA of rats treated with dimethylnitrosamine for a short or a long period. *Chem. Biol. Interac.*, **16**, 301.

OLSEN & LINDAHL. (1980). Methyl group transfer from $O^6$-methylguanine to a protein cysteine residue. *J. Biol. Chem.*, **255**, 10569.

ORTWERTH, B.J. & NOVELLI, G.D. (1969). Studies on the incorporation of L-ethionine-ethyl-1-$^{14}$C into transer RNA of rat liver. *Cancer Res.*, **29**, 380.

PEGG, A. & NICOLL, J.W. (1976). Nitrosamine carcinogenesis: The importance of the persistence in DNA of alkylated bases in the organotropism of tumor induction. In: *Screening Tests in Chemical Carcinogenesis. Vol.* **12.** (Eds. Montesano *et al.*) IARC Scientific Productions. Lyon, p. 571.

PEGG, A.E., ROBERFOID, M., VON BAHR, C. & 5 others. (1982). Removal of $0^6$-methylguanine from DNA by human liver fractions. *Proc. Natl Acad. Sci.*, **79**, 5162.

PFOHL-LESKOWICZ, A., SALAE, C., FUCHS, R.P. & DIRHEIMER, G. (1981). Mechanism of inhibition of enzymatic DNA methylation by 2-(acetylamino) fluorene bound to DNA. *Biochemistry*, **20**, 3020.

POIRIER, L.A., GRANTHOM, P.H. & ROGER, A.E. (1977). The effect of marginally lipotrope-deficient diet on hepatic levels of S-adenosylmethionine and on the urinary metabolites of 2-acetylaminofluorene in rats. *Cancer Res.*, **37**, 744.

POLLACK, Y., STEIN, R., RAZON, A. & CEDAR, H. (1980). Methylation of foreign DNA sequences in eukaroytic cells. *Proc. Natl Acad. Sci.*, **77**, 6463.

REES, L.H. (1975). The biosynthesis of hormones by non-endocrine tumors—a review. *J. Endocrinol.*, **67**, 143.

REGAN, J.D. & SETLOW, R.B. (1974). Two forms of repair in the DNA of human cells damaged by chemical carcinogens and mutagens. *Cancer Res.*, **34**, 3318.

RENARD, A., LEMAITRE, M. & VERLY, W.G. (1982). Repair of $0^6$-alkylguanine lesions in DNA by chromatin enzymes. *Biochimie*, **64**, 803.

RIGGS, A.D. (1975). X-inactivation, differentiation and DNA methylation. *Cytogenet. Cell Genet.*, **14**, 9.

ROBIN, J.C. & NICHOLSON, G.L. (1975). Surfaces of normal and transformed cells. In: *Cancer: A Comprehensive Treatise*, **4.** (Ed. Becker), Plenum: New York, p. 3.

ROGERS, A.E. & NEWBERNE, P.M. (1980).. Lipotrope deficiency in experimental carcinogenesis. *Nutr. Cancer*, **2**, 104.

ROVERA, G. (1975). DNA-Nucleoproteins. In: *Cancer: A Comprehensive Treatise*. **3.** (Ed. Becker) Plenum: New York, p. 405.

RUBERY, E.D. & NEWTON, A.A. (1973). DNA methylation in normal and tumor virus transformed cells in tissue culture. I. The level of DNA methylation in BHK21 cells and in BHK21 cells transformed by polyoma virus ($P_yY$ cells). *Biochem. Biophys. Acta.*, **324**, 24.

SAIKI, J.H., MCCREDIE, K.B., VIETTI, T.S., KEWLETT, J.S., MORRISON, F.S., CASTANYE, J.J., STURKEY, W.S., WHITEAN, J. & HOOGSTRATEN, B. (1978). 5-Azacytidine in acute leukemia. *Cancer*, **42**, 411.

SARMA, D.S.R., RAJALAKSHMI, S. & FARBER, E. (1975). Chemical carcinogenesis: Interactions of carcinogens with nucleic acids. In: *Cancer: A Comprehensive Treatise*, **1.** (Ed. Becker), Plenum: New York, p. 235.

SCARANO, E. (1971). The control of gene function in cell differentiation and in embryogenesis. *Adv. Cytopharmacol.*, **1**, 13.

SHAPIRA, F., DREYFUS, J.C. & SHAPIRA, G. (1963). Anomaly of aldolase in primary liver cancer. *Nature*, **200**, 995.

SINGER, B. (1977). Sites in nucleic acids reacting with alkylating agents of differing carcinogenicity or mutagenicity. *J. Toxicol. Environ. Health*, **2**, 1279.

SINGER, B. (1979). N-nitroso alkylating agents: Formation and persistence of alkyl derivatives in mammalian nucleic acids as contributing factors in carcinogenesis. *J. Natl Cancer Inst.*, **62**, 1329.

SINGER, B., BODELL, W.J., CLEAVER, J.E., THOMAS, G.H., RAJEWSKY, M.F. & THON, W. (1978). Oxygens in DNA are main targets for ethylnitrosourea in normal and xeroderma pigmentosum fibroblasts and fetal rat brain cells. *Nature*, **276**, 85.

SISTA, H.S., LADEN, R.T. & CARUTHERS, M.H. (1979). Studies on gene control regions. The effect of specific adenine-thymine transversions on the lac repressor-lac operator interaction. *Nucleic Acids Res.*, **6**, 2583.

SKIBA, W.E., TAYLOR, M.P., WELLS, M.S., MANGUM, J.H. & AWARD, W.M. (1982). Human hepatic methionine biosynthesis. *J. Biol. Chem.*, **257**, 14994.

SMITH, J.D. & DUNN, D.B. (1959). The occurrence of methylated guanines in ribonucleic acids from several sources. *Biochem. J.*, **72**, 294.

SMITH, R.C. & SALMON, W.D. (1965). Formation of S-adenosylethionine by ethionine-treated rats. *Arch. Biochem. Biophys.*, **111**, 191.

SRINIVASAN, P.R. & BOREK, E. (1964). Enzymatic alteration of nucleic acid structures. *Science*, **145**, 548.

STEKOL, J.A. (1955). Synthetic pathways of methionine, cysteine and threonine. In: *Amino Acid Metabolism*. (Eds. McElroy & Glass), The Johns Hopkins Press: Baltimore, p. 509.

STEKOL, J.A. (1963). Biochemical basis for ethionine effects on tissues. *Adv. Enzymol.*, **25**, 369.

STONER, G.D., SHIMKIN, M.B., KNEAZEFF, A.J., WEISBURGER, J.H., WEISBURGER, E.K. & GORI, G.B. (1973). Test for carcinogeneicity of food additives and chemotherapeutic agents by the pulmonary tumor response in strain A mice. *Cancer Res.*, **33**, 3069.

SUTTER, D. & DOERFLER, W. (1980). Methylation of integrated adenovirus type 12 DNA sequences in transformed cells is inversely correlated with viral gene expression. *Proc. Natl Acad. Sci.*, **77**, 253.

SWANN, P.F., PEGG, A.B., HAWKS, A.E., FARBER, E. & MAGEE, P.N. (1971). Evidence for ethylation of rat liver DNA after administration of ethionine. *Biochem. J.*, **123**, 175.

TAKEBE, H., MIKI, Y., KOZAKA, T., FURUYAMA, D.I., TANSKA, K., SASAKI, M.S., FUJIWARA, Y. & AKIBA, H. (1977). DNA repair characteristics and skin cancers of xeroderma pigmentosum patients in Japan. *Cancer Res.*, **37**, 490.

VANYUSHIN, B.F., ZIN'KOVSKAYA, G.G. & BERDYSHEV, G.D. (1980). Decrease of DNA methylation level in cattle due to aging. *Molekuyarnaya Biol.*, **14**, 857.

VOGLER, W.R., MILLER, D.S. & KELLER, J.W. (1976). 5-Azacytidine (NSC 102816): A new drug for the treatment of myeloblastic leukemia. *Blood*, **48**, 331.

VON HOFF, D.D., SLAVIK, M. & MUGGIA, F.M. (1976). 5-Azacytidine. A new anticancer drug with effectiveness in acute myelogenous leukemia. *Ann. Intern. Med.*, **85**, 237.

WAECHTER, D.E. & BASERGA, R. (1982). Effect of methylation on expression of microinjected genes. *Proc. Natl Acad. Sci.*, **79**, 1106.

WALDSTEIN, E.A., CAO, E.-H. & SETLOW, R.B. (1982). Adaptive resynthesis of $0^6$-methylguanine-accepting protein can explain the differences between mammalian cells proficient and deficient in methyl excision repair. *Proc. Natl Acad. Sci.*, **79**, 5117.

WEINBERG, A.G., MIZE, C.E. & WORTHEN, H.G. (1976). The occurrence of hepatoma in the chronic form of hereditary tyrosinemia. *J. Pediatrics*, **88**, 434.

WEINBERG, R.A. (1982). Oncogenes of spontaneous and chemically-induced tumors. *Adv. Cancer Res.*, **36**, 149.

WEINHOUSE, S. (1970). Respiration glycolysis, and enzyme alterations in liver neoplasms. Miami Winter Symposium 2, 462.

WEINHOUSE, S., SHATTON, J.B., CRISS, W.E., FARINA, F.A. & MORRIS, H.P. (1972). Isoenzymes in relation to differentiation in transplantable rat hepatomas. *Gann Monog. Cancer Res.*, **13**, 1.

WEINHOUSE, S. (1974). Enzymatic and metabolic alterations in experimental hepatomas. In: *Differentiation and Control of Malignancy in Tumor Cells*. (Ed. Nakahara), University Park Press: Tokyo, p. 187.

WEINHOUSE, S. (1980). New dimensions in the biology of cancer. *Cancer*, **45**, 2975.

YUAN, R. & MESELSON, M. (1970). A specific complex between a restriction endonuclease and its DNA substrate. *Proc. Natl Acad. Sci.*, **65**, 357.


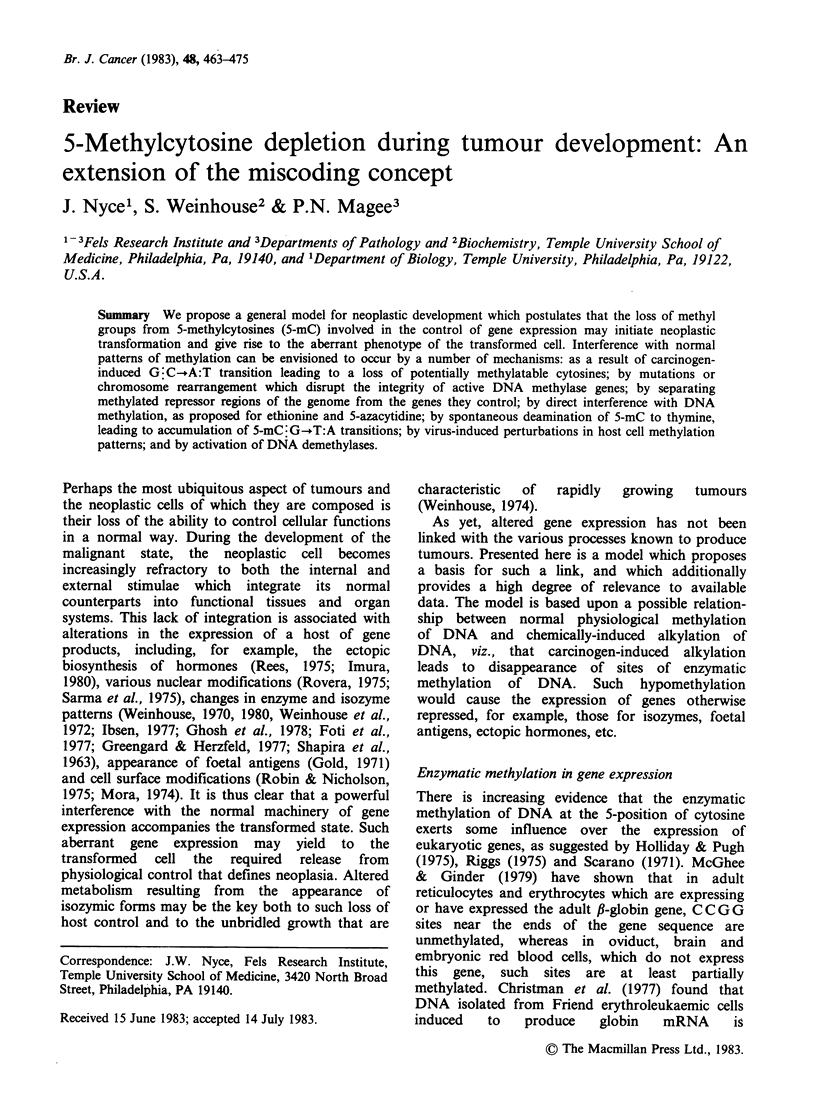

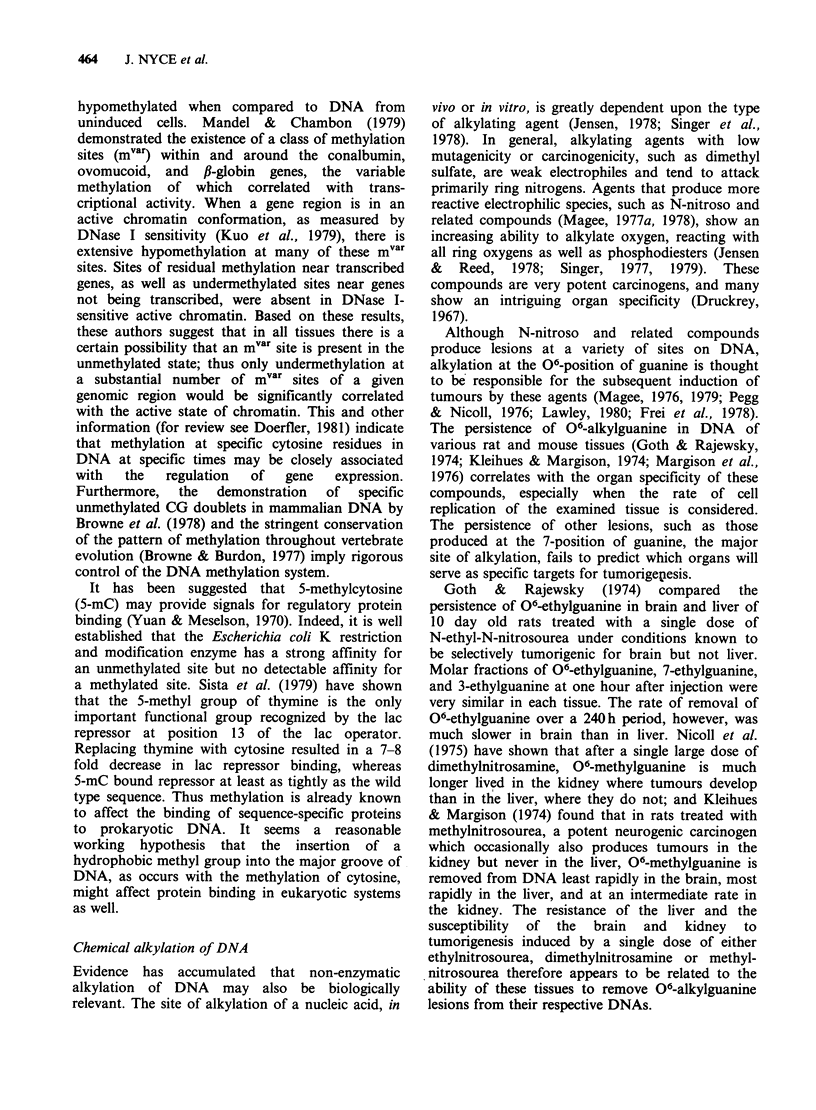

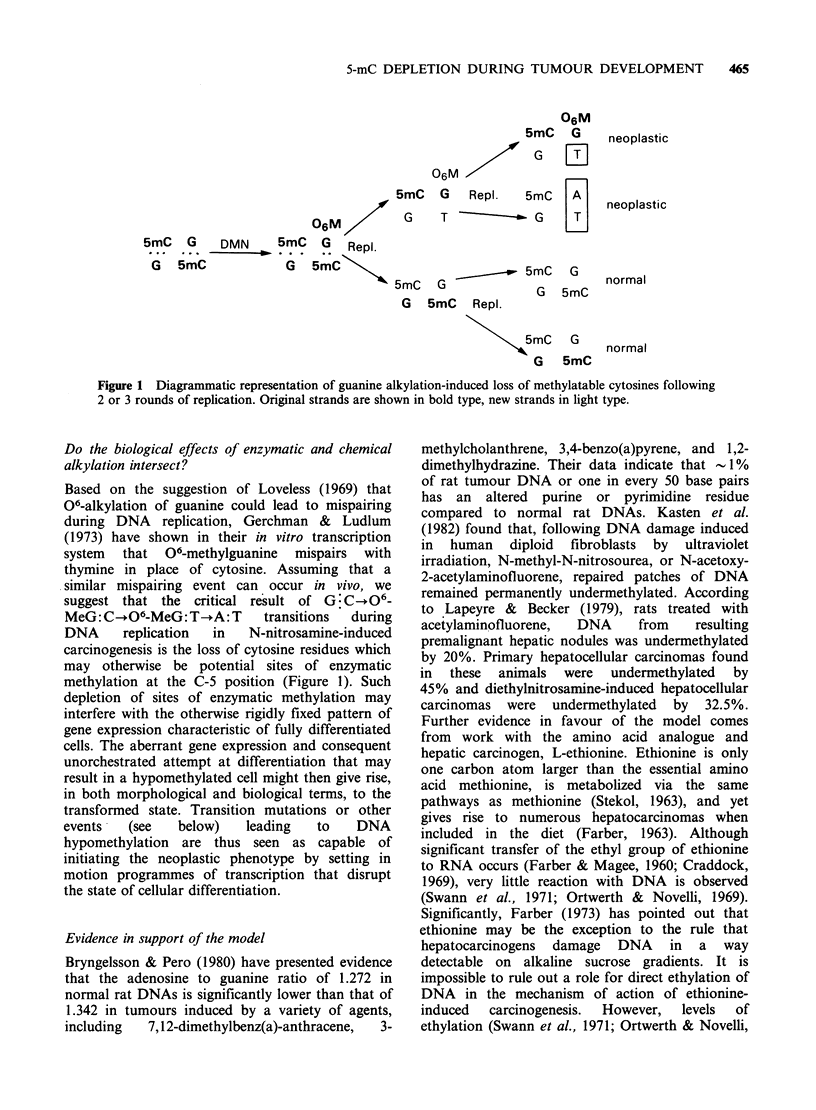

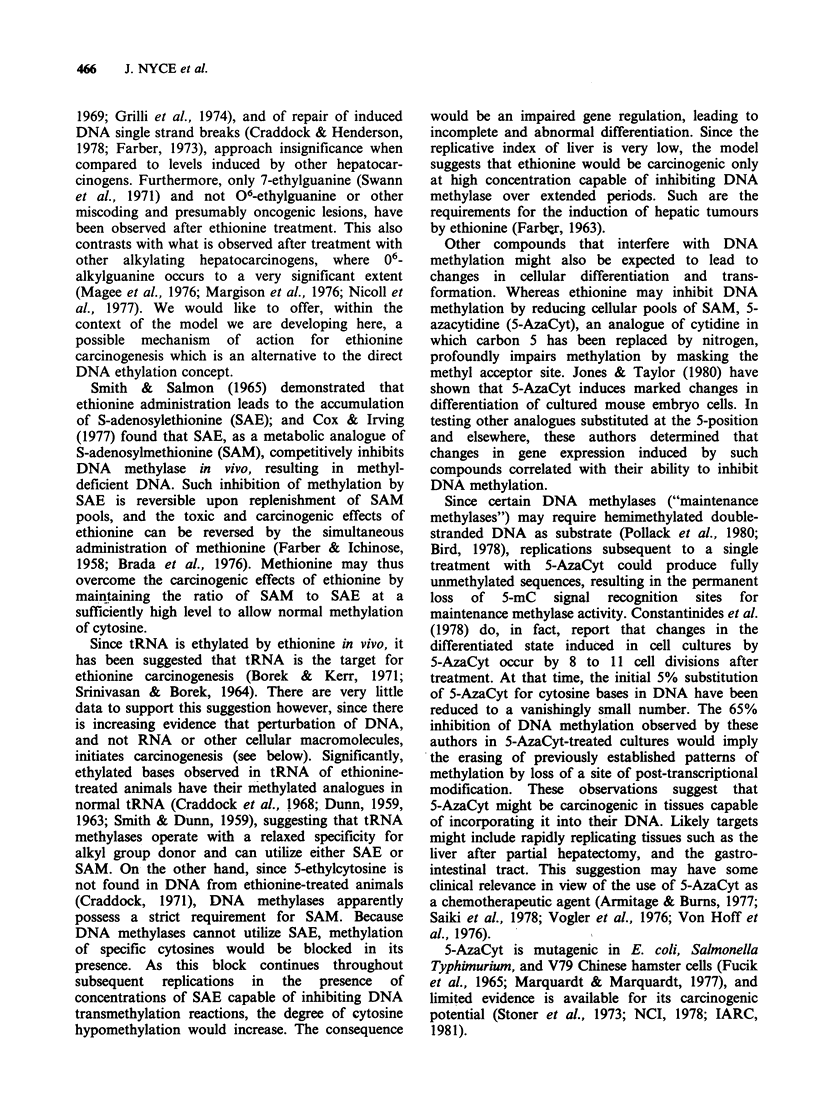

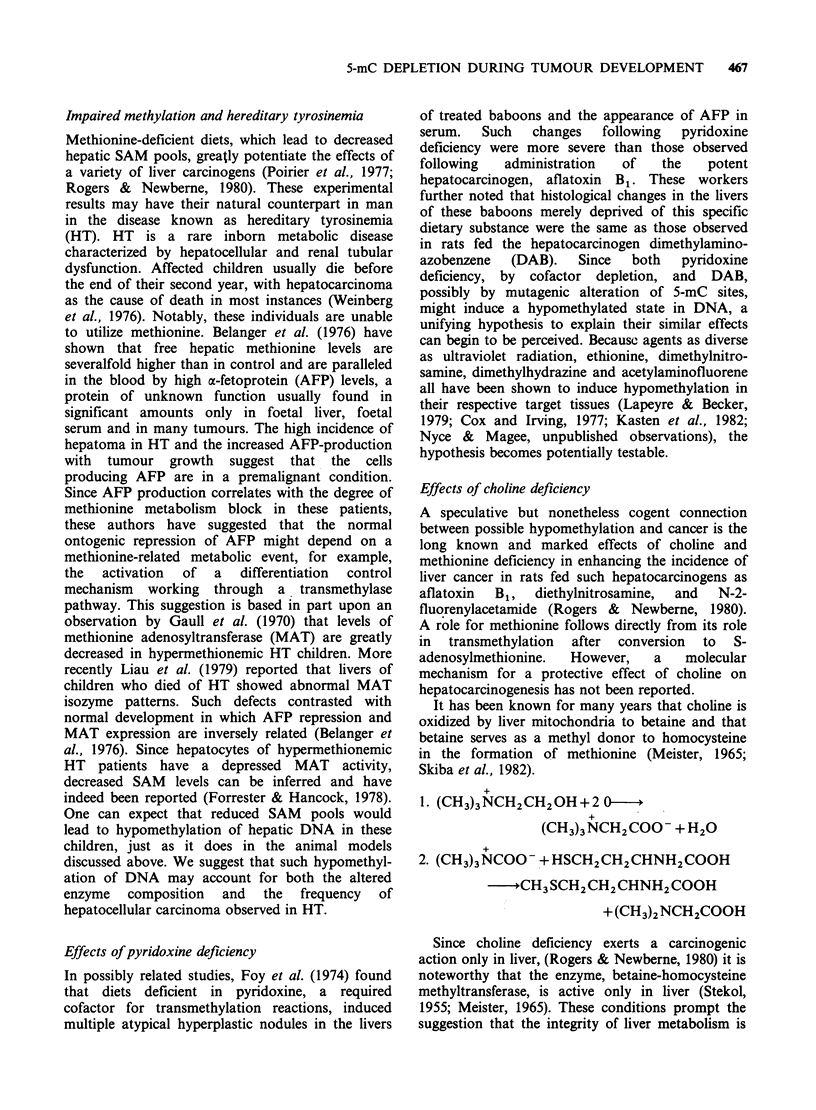

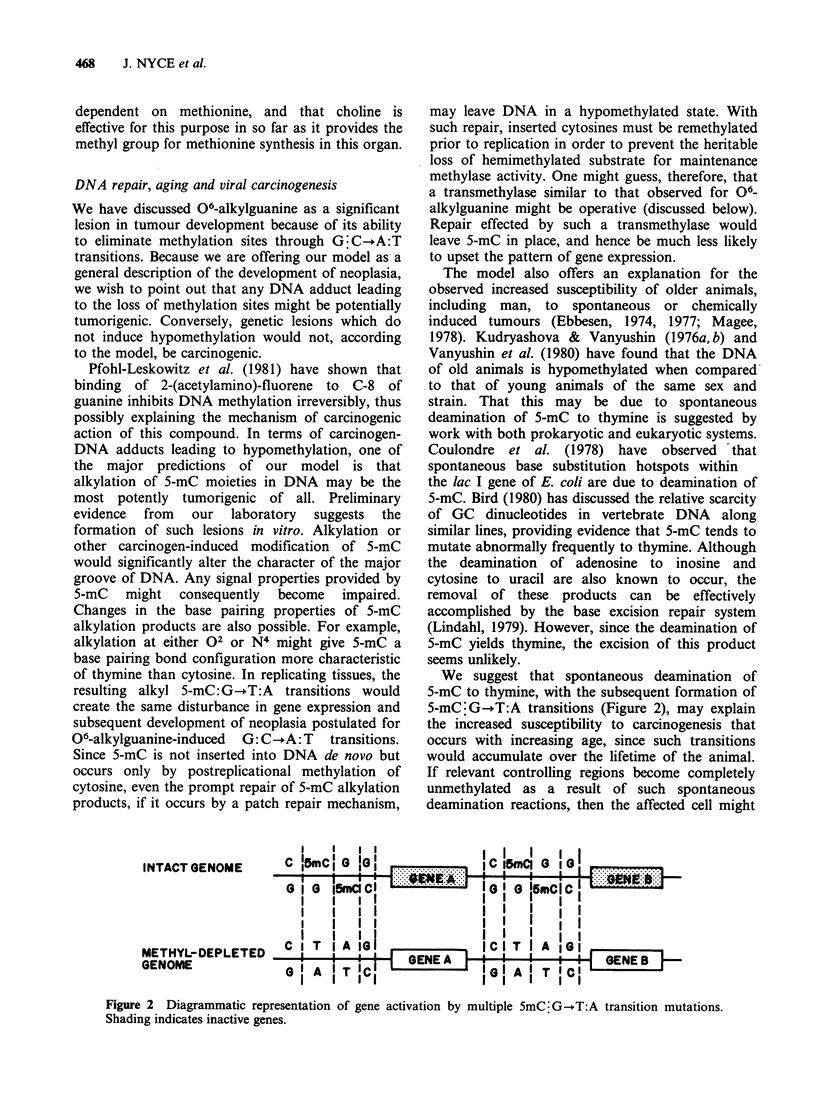

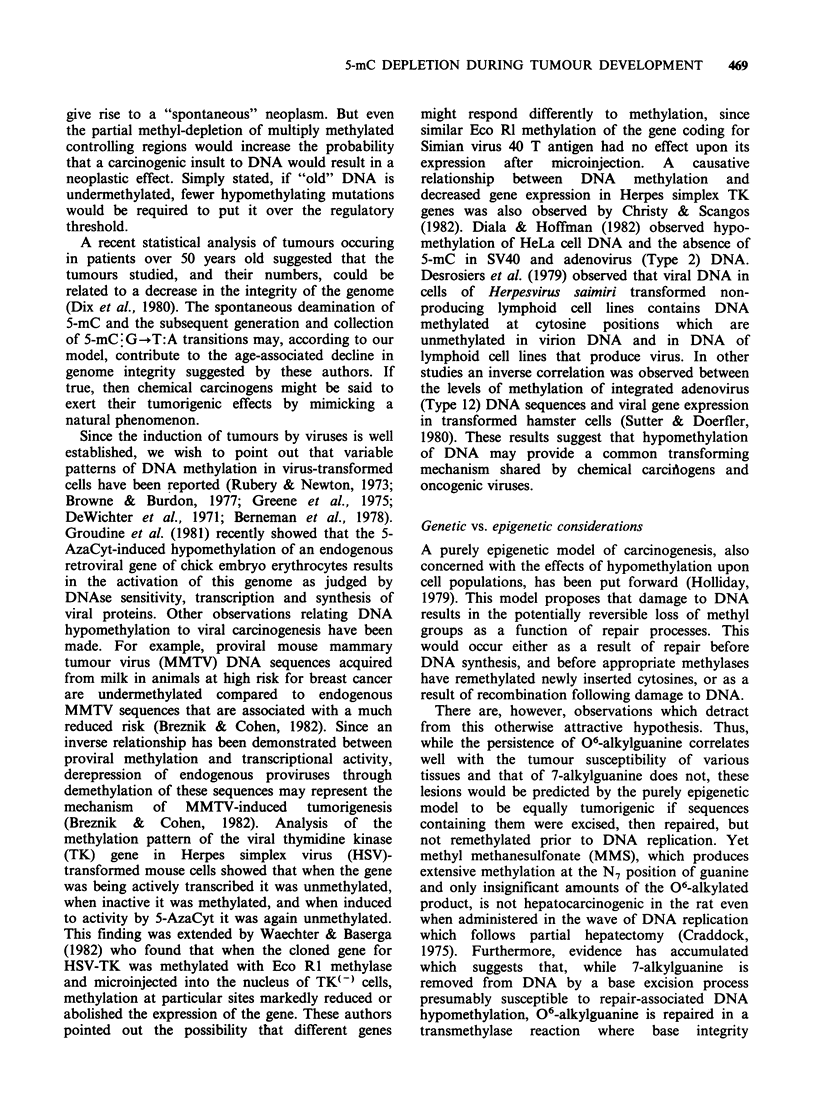

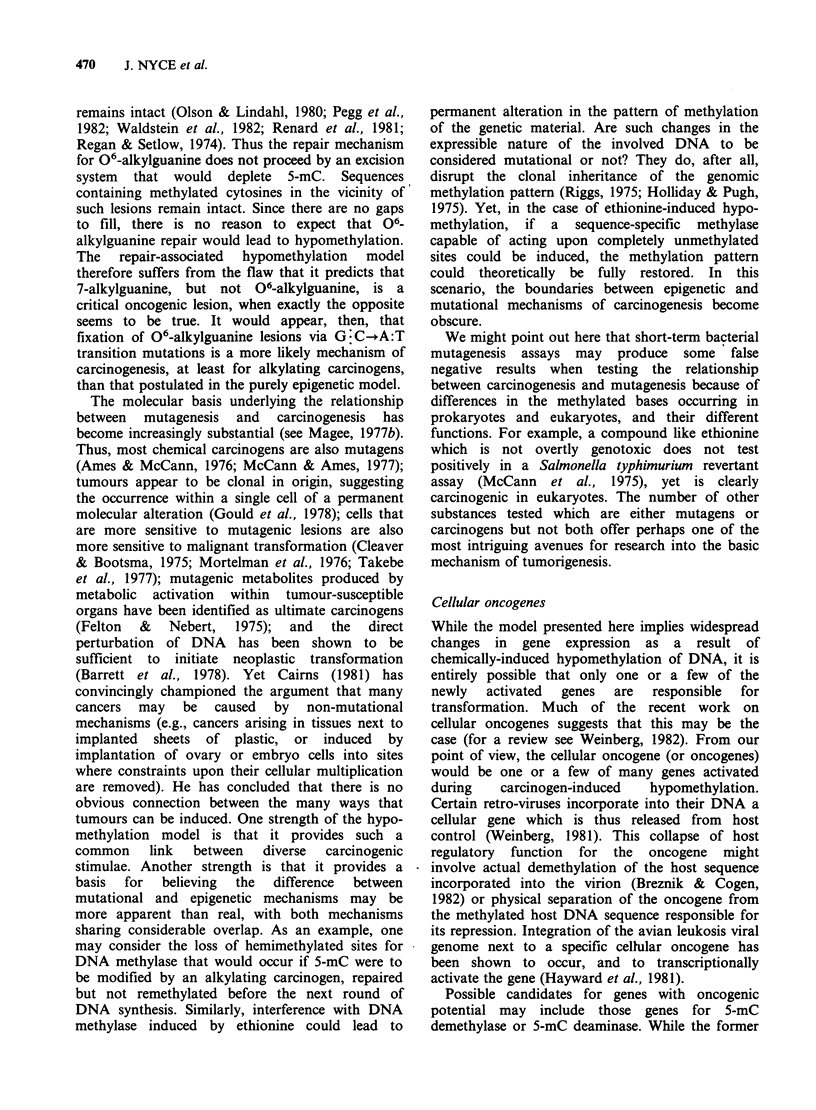

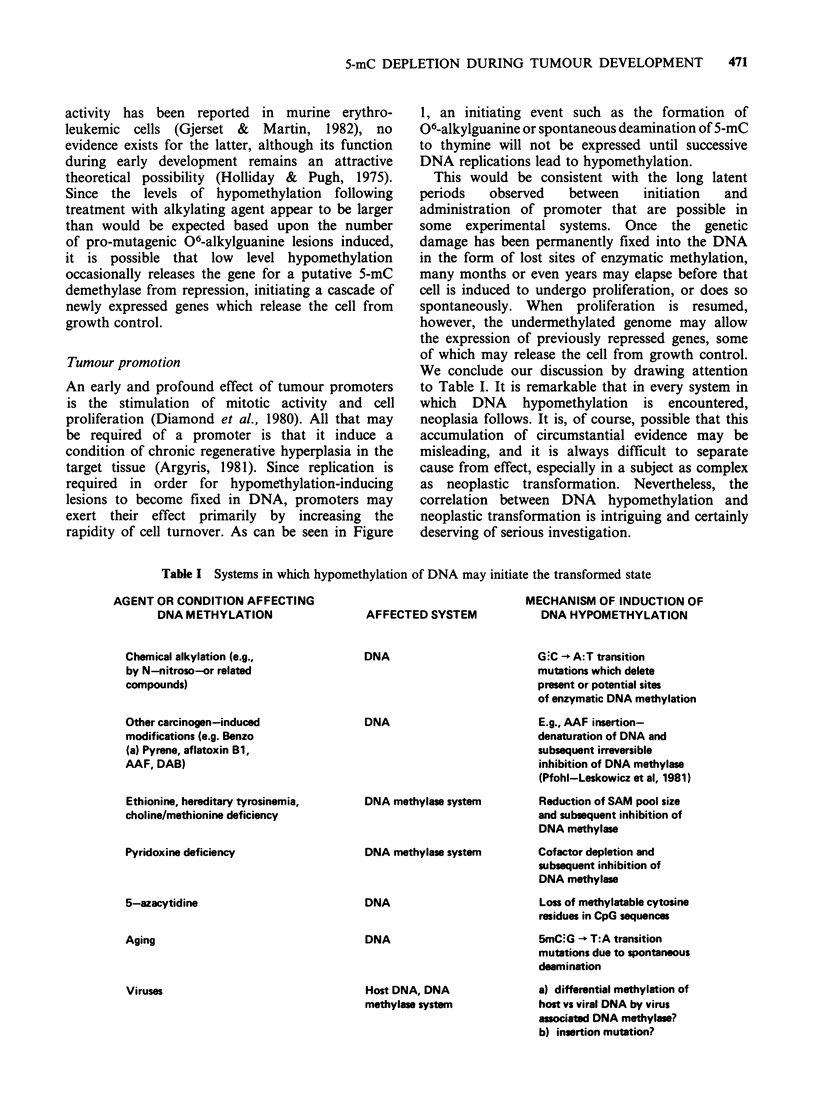

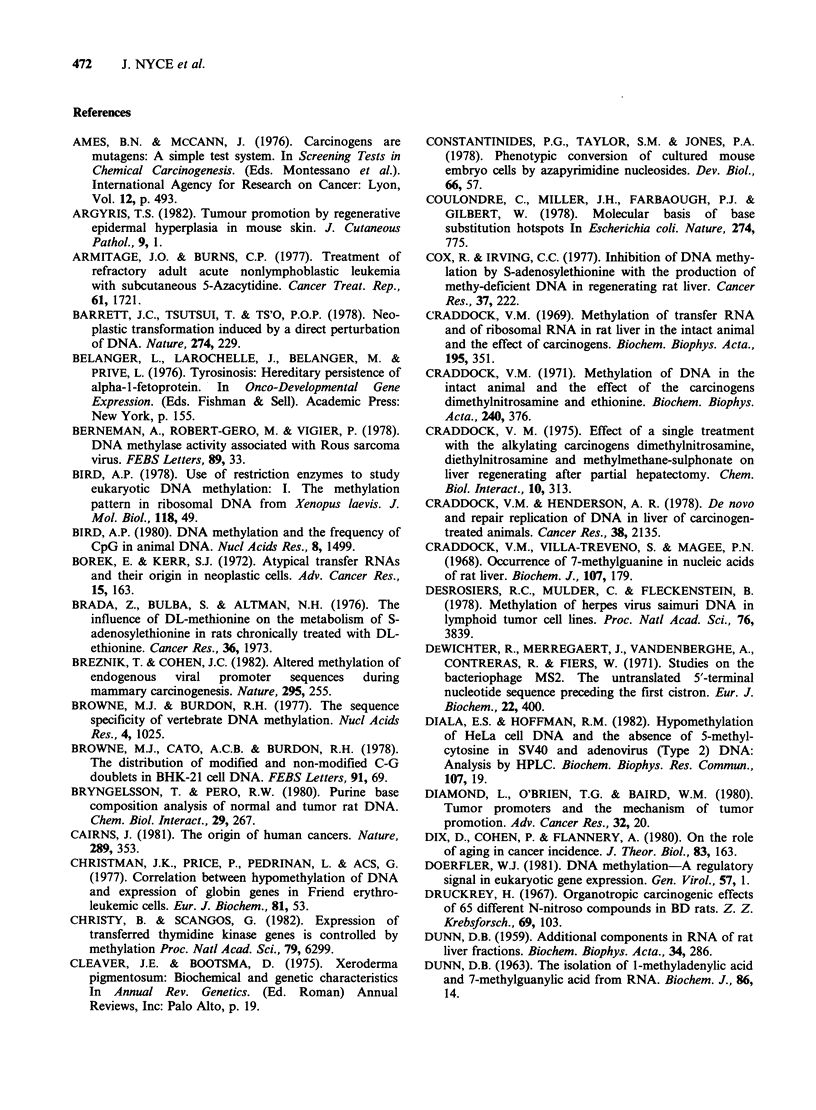

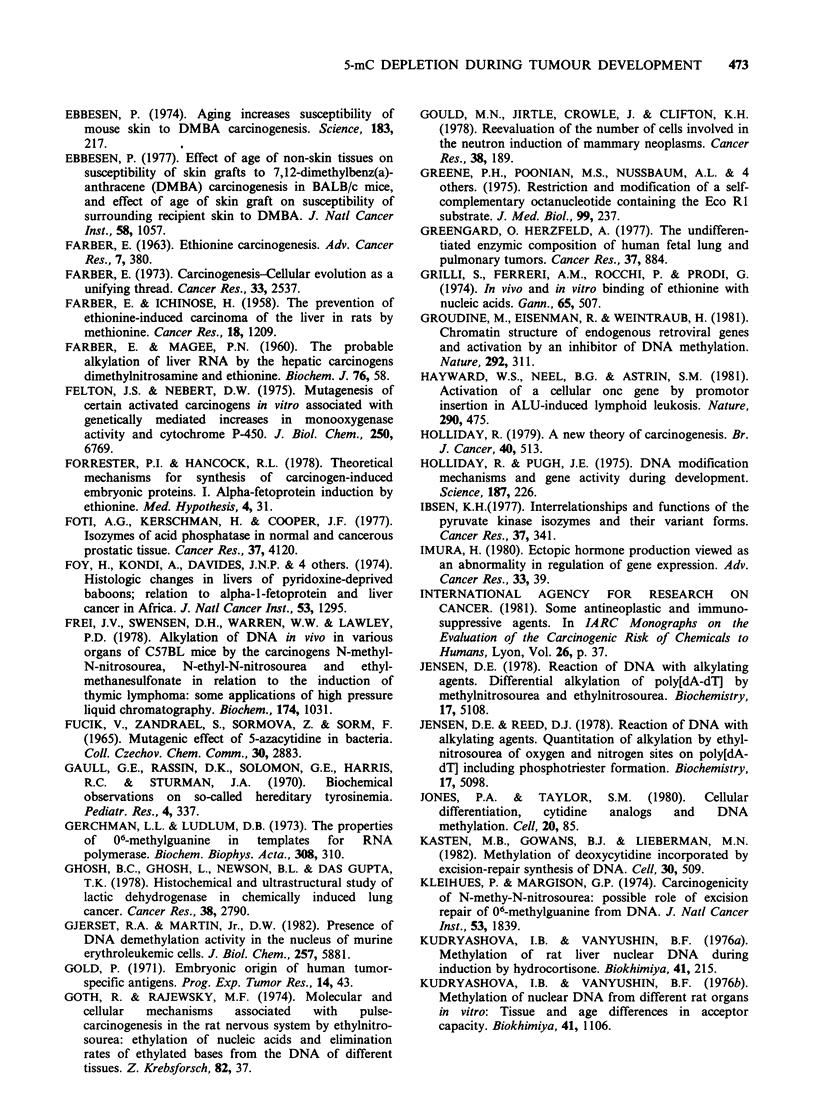

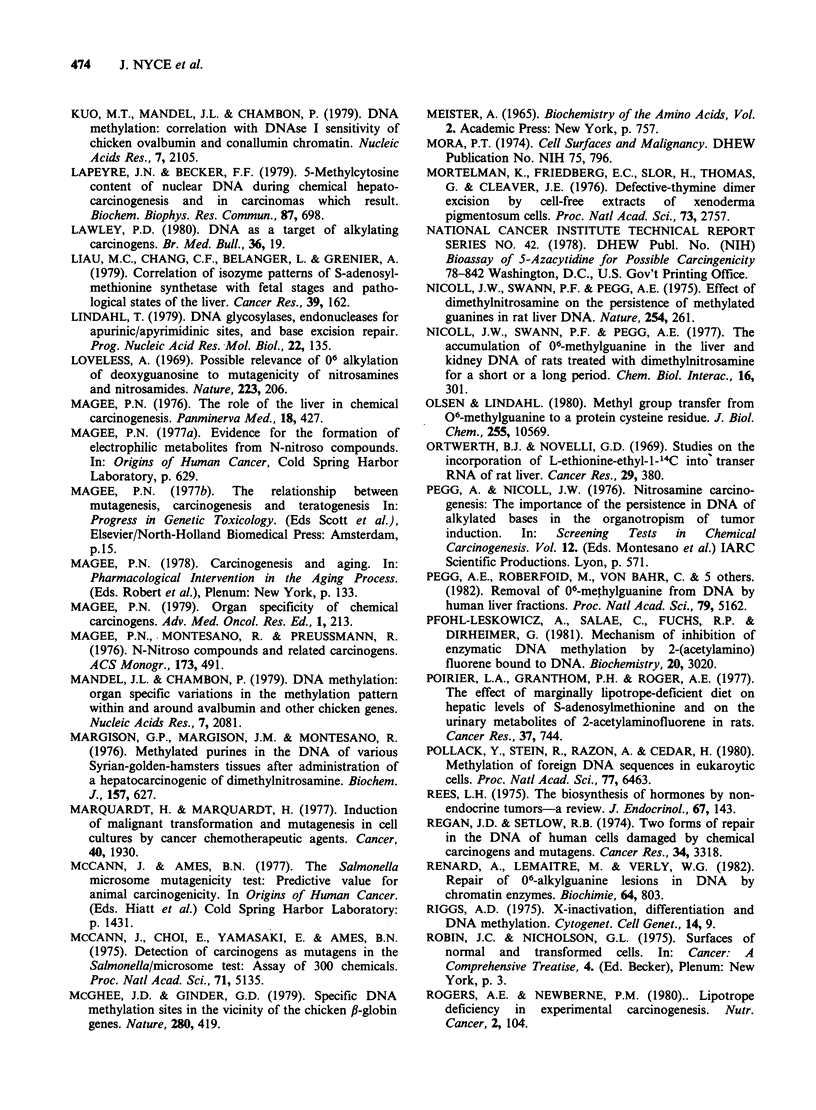

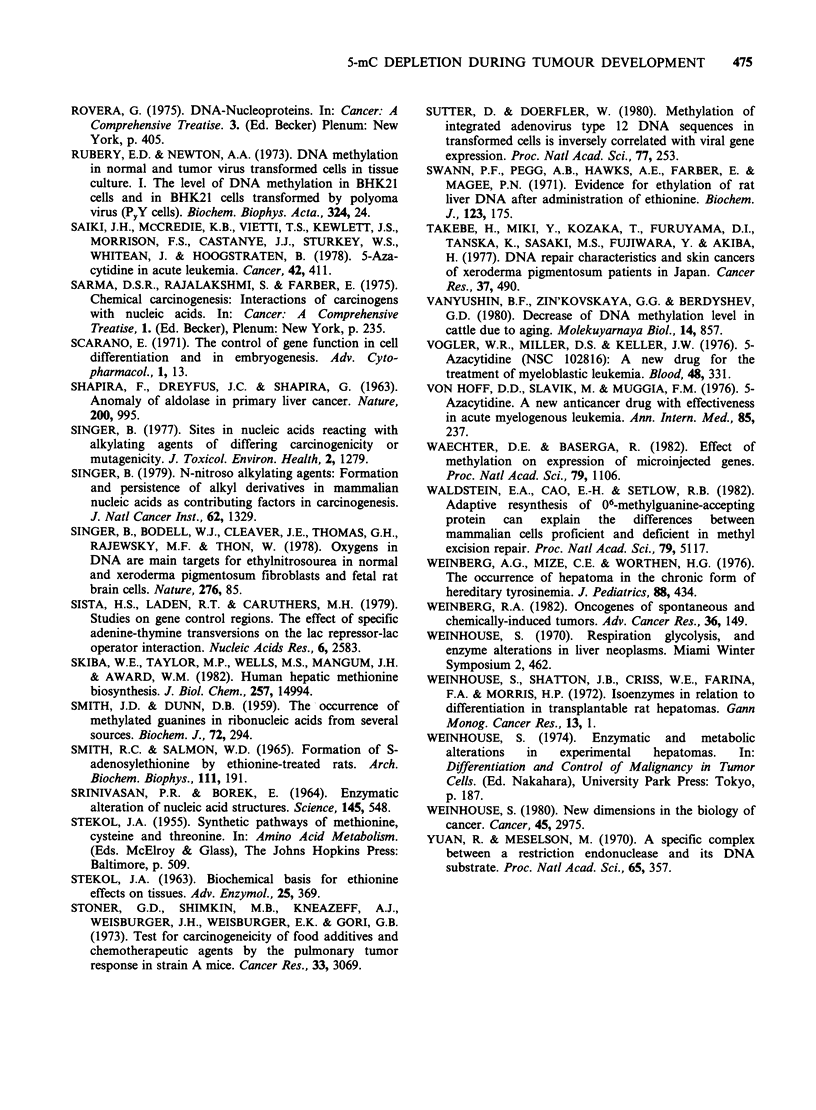


## References

[OCR_01082] Argyris T. S. (1982). Tumor promotion by regenerative epidermal hyperplasia in mouse skin.. J Cutan Pathol.

[OCR_01087] Armitage J. O., Burns C. P. (1977). Treatment of refractory adult acute nonlymphoblastic leukemia with subcutaneous 5-azacytidine.. Cancer Treat Rep.

[OCR_01093] Barrett J. C., Tsutsui T., Ts'o P. O. (1978). Neoplastic transformation induced by a direct perturbation of DNA.. Nature.

[OCR_01105] Berneman A., Robert-Gero M., Vigier P. (1978). DNA methylase activity associated with Rous sarcoma virus.. FEBS Lett.

[OCR_01116] Bird A. P. (1980). DNA methylation and the frequency of CpG in animal DNA.. Nucleic Acids Res.

[OCR_01110] Bird A. P. (1978). Use of restriction enzymes to study eukaryotic DNA methylation: II. The symmetry of methylated sites supports semi-conservative copying of the methylation pattern.. J Mol Biol.

[OCR_01120] Borek E., Kerr S. J. (1972). Atypical transfer RNA's and their origin in neoplastic cells.. Adv Cancer Res.

[OCR_01131] Breznik T., Cohen J. C. (1982). Altered methylation of endogenous viral promoter sequences during mammary carcinogenesis.. Nature.

[OCR_01136] Browne M. J., Burdon R. H. (1977). The sequence specificity of vertebrate DNA methylation.. Nucleic Acids Res.

[OCR_01141] Browne M. J., Cato A. C., Burdon R. H. (1978). The distribution of modified and non-modified C-G doublets in BHK-21 cell DNA.. FEBS Lett.

[OCR_01146] Bryngelsson T., Pero R. W. (1980). Purine base composition analysis of normal and tumor rat DNA.. Chem Biol Interact.

[OCR_01151] Cairns J. (1981). The origin of human cancers.. Nature.

[OCR_01155] Christman J. K., Price P., Pedrinan L., Acs G. (1977). Correlation between hypomethylation of DNA and expression of globin genes in Friend erythroleukemia cells.. Eur J Biochem.

[OCR_01161] Christy B., Scangos G. (1982). Expression of transferred thymidine kinase genes is controlled by methylation.. Proc Natl Acad Sci U S A.

[OCR_01172] Constantinides P. G., Taylor S. M., Jones P. A. (1978). Phenotypic conversion of cultured mouse embryo cells by aza pyrimidine nucleosides.. Dev Biol.

[OCR_01178] Coulondre C., Miller J. H., Farabaugh P. J., Gilbert W. (1978). Molecular basis of base substitution hotspots in Escherichia coli.. Nature.

[OCR_01184] Cox R., Irving C. C. (1977). Inhibition of DNA methylation by S-adenosylethionine with the production of methyl-deficient DNA in regenerating rat liver.. Cancer Res.

[OCR_01202] Craddock V. M. (1975). Effect of a single treatment with the alkylating carcinogens dimethynitrosamine, diethylnitrosamine and methyl methanesulphonate, on liver regenerating after partial hepatectomy. I. Test for induction of liver carcinomas.. Chem Biol Interact.

[OCR_01209] Craddock V. M., Henderson A. R. (1978). De novo and repair replication of DNA in liver of carcinogen-treated animals.. Cancer Res.

[OCR_01190] Craddock V. M. (1969). Methylation of transfer RNA and of ribosomal RNA in rat liver in the intact animal and the effect of carcinogens.. Biochim Biophys Acta.

[OCR_01214] Craddock V. M., Villa-Trevino S., Magee P. N. (1968). Occurrence of 7-methylguanine in nucleic acids of rat liver.. Biochem J.

[OCR_01257] DUNN D. B. (1959). Additional components in ribonucleic acid of rat-liver fractions.. Biochim Biophys Acta.

[OCR_01219] Desrosiers R. C., Mulder C., Fleckenstein B. (1979). Methylation of Herpesvirus saimiri DNA in lymphoid tumor cell lines.. Proc Natl Acad Sci U S A.

[OCR_01232] Diala E. S., Hoffman R. M. (1982). Hypomethylation of HeLa cell DNA and the absence of 5-methylcytosine in SV40 and adenovirus (type 2) DNA: analysis by HPLC.. Biochem Biophys Res Commun.

[OCR_01244] Dix D., Cohen P., Flannery J. (1980). On the role of aging in cancer incidence.. J Theor Biol.

[OCR_01248] Doerfler W. (1981). DNA methylation--a regulatory signal in eukaryotic gene expression.. J Gen Virol.

[OCR_01252] Druckrey H., Preussmann R., Ivankovic S., Schmähl D. (1967). Organotrope carcinogene Wirkungen bei 65 verschiedenen N-Nitroso-Verbindungen an BD-Ratten.. Z Krebsforsch.

[OCR_01268] Ebbesen P. (1974). Aging increases susceptibility of mouse skin to DMBA carcinogenesis independent of general immune status.. Science.

[OCR_01273] Ebbesen P. (1977). Effect of age of non-skin tissues on susceptibility of skin grafts to 7,12-dimethylbenz[alpha]anthracene (DMBA) carcinogenesis in BALB/c mice, and effect of age of skin graft on susceptibility of surrounding recipient skin to DMBA.. J Natl Cancer Inst.

[OCR_01289] FARBER E., ICHINOSE H. (1958). The prevention of ethionine-induced carcinoma of the liver in rats by methionine.. Cancer Res.

[OCR_01285] Farber E. (1973). Carcinogenesis--cellular evolution as a unifying thread: Presidential address.. Cancer Res.

[OCR_01299] Felton J. S., Nebert D. W. (1975). Mutagenesis of certain activated carcinogens in vitro associated with genetically mediated increases in monooxygenase activity and cytochrome P 1-450.. J Biol Chem.

[OCR_01306] Forrester P. I., Hancock R. L. (1978). Theoretical mechanisms for synthesis of carcinogen-induced embryonic proteins. I. Alpha-fetoprotein induction by ethionine.. Med Hypotheses.

[OCR_01312] Foti A. G., Herschman H., Cooper J. F. (1977). Isozymes of acid phosphatase in normal and cancerous human prostatic tissue.. Cancer Res.

[OCR_01317] Foy H., Kondi A., Davies J. N., Anderson B., Parker A., Preston J., Peers F. G. (1974). Histologic changes in livers of pyridoxine-deprived baboons--relation to alpha1 fetoprotein and liver cancer in Africa.. J Natl Cancer Inst.

[OCR_01323] Frei J. V., Swenson D. H., Warren W., Lawley P. D. (1978). Alkylation of deoxyribonucleic acid in vivo in various organs of C57BL mice by the carcinogens N-methyl-N-nitrosourea, N-ethyl-N-nitrosourea and ethyl methanesulphonate in relation to induction of thymic lymphoma. Some applications of high-pressure liquid chromatography.. Biochem J.

[OCR_01337] Gaull G. E., Rassin D. K., Solomon G. E., Harris R. C., Sturman J. A. (1970). Biochemical observations on so-called hereditary tyrosinemia.. Pediatr Res.

[OCR_01343] Gerchman L. L., Ludlum D. B. (1973). The properties of O 6 -methylguanine in templates for RNA polymerase.. Biochim Biophys Acta.

[OCR_01348] Ghosh B. C., Ghosh L., Newson B. L., Das Gupta T. K. (1978). Histochemical and ultrastructural study of lactic dehydrogenase in chemically induced lung cancer.. Cancer Res.

[OCR_01363] Goth R., Rajewsky M. F. (1974). Molecular and cellular mechanisms associated with pulse-carcinogenesis in the rat nerbous system by ethyinitrosourea: ethylation of nucleic acids and elimination rates of ethylated bases from the DNA of different tissues.. Z Krebsforsch Klin Onkol Cancer Res Clin Oncol.

[OCR_01373] Gould M. N., Jirtle R., Crowley J., Clifton K. H. (1978). Reevaluation of the number of cells involved in the neutron induction of mammary neoplasms.. Cancer Res.

[OCR_01377] Greene P. H., Poonian M. S., Nussbaum A. L., Tobias L., Garfin D. E., Boyer H. W., Goodman H. M. (1975). Restriction and modification of a self-complementary octanucleotide containing the EcoRI substrate.. J Mol Biol.

[OCR_01383] Greengard O., Herzfeld A. (1977). The undifferentiated enzymic composition of human fetal lung and pulmonary tumors.. Cancer Res.

[OCR_01388] Grilli S., Ferreri A. M., Rocchi P., Prodi G. (1974). In vivo and in vitro binding of ethionine with nucleic acids.. Gan.

[OCR_01393] Groudine M., Eisenman R., Weintraub H. (1981). Chromatin structure of endogenous retroviral genes and activation by an inhibitor of DNA methylation.. Nature.

[OCR_01399] Hayward W. S., Neel B. G., Astrin S. M. (1981). Activation of a cellular onc gene by promoter insertion in ALV-induced lymphoid leukosis.. Nature.

[OCR_01405] Holliday R. (1979). A new theory of carcinogenesis.. Br J Cancer.

[OCR_01409] Holliday R., Pugh J. E. (1975). DNA modification mechanisms and gene activity during development.. Science.

[OCR_01414] Ibsen K. H. (1977). Interrelationships and functions of the pyruvate kinase isozymes and their variant forms: a review.. Cancer Res.

[OCR_01419] Imura H. (1980). Ectopic hormone production viewed as an abnormality in regulation of gene expression.. Adv Cancer Res.

[OCR_01431] Jensen D. E. (1978). Reaction of DNA with alkylating agents. Differential alkylation of poly[dA-dT[ by methylnitrosourea and ethylnitrosourea.. Biochemistry.

[OCR_01437] Jensen D. E., Reed D. J. (1978). Reaction of DNA with alkylating agents. Quantitation of alkylation by ethylnitrosourea of oxygen and nitrogen sites on poly[dA-dT] including phosphotriester formation.. Biochemistry.

[OCR_01444] Jones P. A., Taylor S. M. (1980). Cellular differentiation, cytidine analogs and DNA methylation.. Cell.

[OCR_01449] Kastan M. B., Gowans B. J., Lieberman M. W. (1982). Methylation of deoxycytidine incorporated by excision-repair synthesis of DNA.. Cell.

[OCR_01454] Kleihues P., Margison G. P. (1974). Carcinogenicity of N-methyl-N-nitrosourea: possible role of excision repair of O6-methylguanine from DNA.. J Natl Cancer Inst.

[OCR_01460] Kudriashova I. B., Vaniushin B. F. (1976). Metilirovanie iadernoi DNK pecheni krysy pri induktsii gidokortizonom. Biokhimiia.

[OCR_01465] Kudriashova I. B., Vaniushin B. F. (1976). Metilirovanie in vitro iadernoi DNK iz raznykh organov krysy: Tkanevye i vozrastnye razlichiia aktseptornoi sposobnosti DNK.. Biokhimiia.

[OCR_01473] Kuo M. T., Mandel J. L., Chambon P. (1979). DNA methylation: correlation with DNase I sensitivity of chicken ovalbumin and conalbumin chromatin.. Nucleic Acids Res.

[OCR_01479] Lapeyre J. N., Becker F. F. (1979). 5-Methylcytosine content of nuclear DNA during chemical hepatocarcinogenesis and in carcinomas which result.. Biochem Biophys Res Commun.

[OCR_01485] Lawley P. D. (1980). DNA as a target of alkylating carcinogens.. Br Med Bull.

[OCR_01489] Liau M. C., Chang C. F., Belanger L., Grenier A. (1979). Correlation of isozyme patterns of S-adenosylmethionine synthetase with fetal stages and pathological states of the liver.. Cancer Res.

[OCR_01495] Lindahl T. (1979). DNA glycosylases, endonucleases for apurinic/apyrimidinic sites, and base excision-repair.. Prog Nucleic Acid Res Mol Biol.

[OCR_01500] Loveless A. (1969). Possible relevance of O-6 alkylation of deoxyguanosine to the mutagenicity and carcinogenicity of nitrosamines and nitrosamides.. Nature.

[OCR_01505] Magee P. N. (1976). The role of the liver in chemical carcinogenesis.. Panminerva Med.

[OCR_01536] Mandel J. L., Chambon P. (1979). DNA methylation: organ specific variations in the methylation pattern within and around ovalbumin and other chicken genes.. Nucleic Acids Res.

[OCR_01542] Margison G. P., Margison J. M., Montesano R. (1976). Methylated purines in the deoxyribonucleic acid of various Syrian-golden-hamster tissues after administration of a hepatocarcinogenic dose of dimethylnitrosamine.. Biochem J.

[OCR_01549] Marquardt H., Marquardt H. (1977). Induction of malignant transformation and mutagenesis in cell cultures by cancer chemotherapeutic agents.. Cancer.

[OCR_01562] McCann J., Choi E., Yamasaki E., Ames B. N. (1975). Detection of carcinogens as mutagens in the Salmonella/microsome test: assay of 300 chemicals.. Proc Natl Acad Sci U S A.

[OCR_01568] McGhee J. D., Ginder G. D. (1979). Specific DNA methylation sites in the vicinity of the chicken beta-globin genes.. Nature.

[OCR_01581] Mortelmans K., Friedberg E. C., Slor H., Thomas G., Cleaver J. E. (1976). Defective thymine dimer excision by cell-free extracts of xeroderma pigmentosum cells.. Proc Natl Acad Sci U S A.

[OCR_01593] Nicoll J. W., Swann P. F., Pegg A. E. (1975). Effect of dimethylnitrosamine on persistence of methylated guanines in rat liver and kidney DNA.. Nature.

[OCR_01598] Nicoll J. W., Swann P. F., Pegg A. E. (1977). The accumulation of O6-methylguanine in the liver and kidney DNA of rats treated with dimethylinitrosamine for a short or a long period.. Chem Biol Interact.

[OCR_01605] Olsson M., Lindahl T. (1980). Repair of alkylated DNA in Escherichia coli. Methyl group transfer from O6-methylguanine to a protein cysteine residue.. J Biol Chem.

[OCR_01610] Ortwerth B. J., Novelli G. D. (1969). Studies on the incorporation of L-ethionine-ethyl-l-14C into the transfer RNA of rat liver.. Cancer Res.

[OCR_01623] Pegg A. E., Roberfroid M., von Bahr C., Foote R. S., Mitra S., Bresil H., Likhachev A., Montesano R. (1982). Removal of O6-methylguanine from DNA by human liver fractions.. Proc Natl Acad Sci U S A.

[OCR_01628] Pfohl-Leszkowicz A., Salas C., Fuchs R. P., Dirheimer G. (1981). Mechanism of inhibition of enzymatic deoxyribonucleic acid methylation by 2-(acetylamino)fluorene bound to deoxyribonucleic acid.. Biochemistry.

[OCR_01634] Poirier L. A., Grantham P. H., Rogers A. E. (1977). The effects of a marginally lipotrope-deficient diet on the hepatic levels of S-adenosylmethionine and on the urinary metabolites of 2-acetylaminofluorene in rats.. Cancer Res.

[OCR_01641] Pollack Y., Stein R., Razin A., Cedar H. (1980). Methylation of foreign DNA sequences in eukaryotic cells.. Proc Natl Acad Sci U S A.

[OCR_01646] Rees L. H. (1975). The biosynthesis of hormones by non-endocrine tumours--a review.. J Endocrinol.

[OCR_01650] Regan J. D., Setlow R. B. (1974). Two forms of repair in the DNA of human cells damaged by chemical carcinogens and mutagens.. Cancer Res.

[OCR_01655] Renard A., Lemaitre M., Verly W. G. (1982). Repair of O6-alkylguanine lesions in DNA by chromatin enzymes.. Biochimie.

[OCR_01660] Riggs A. D. (1975). X inactivation, differentiation, and DNA methylation.. Cytogenet Cell Genet.

[OCR_01682] Rubery E. D., Newton A. A. (1973). DNA methylation in normal and tumour virus-transformed cells in tissue culture. I. The level of DNA methylation in BHK21 cells and in BHK21 cells transformed by polyoma virus (PyY cells).. Biochim Biophys Acta.

[OCR_01706] SCHAPIRA F., DREYFUS J. C., SCHAPIRA G. (1963). ANOMALY OF ALDOLASE IN PRIMARY LIVER CANCER.. Nature.

[OCR_01740] SMITH J. D., DUNN D. B. (1959). The occurrence of methylated guanines in ribonucleic acids from several sources.. Biochem J.

[OCR_01750] SRINIVASAN P. R., BOREK E. (1964). ENZYMATIC ALTERATION OF NUCLEIC ACID STRUCTURE.. Science.

[OCR_01760] STEKOL J. A. (1963). BIOCHEMICAL BASIS FOR ETHIONINE EFFECTS ON TISSUES.. Adv Enzymol Relat Areas Mol Biol.

[OCR_01701] Scarano E. (1971). The control of gene function in cell differentiation and in embryogenesis.. Adv Cytopharmacol.

[OCR_01722] Singer B., Bodell W. J., Cleaver J. E., Thomas G. H., Rajewsky M. F., Thon W. (1978). Oxygens in DNA are main targets for ethylnitrosourea in normal and xeroderma pigmentosum fibroblasts and fetal rat brain cells.. Nature.

[OCR_01716] Singer B. (1979). N-nitroso alkylating agents: formation and persistence of alkyl derivatives in mammalian nucleic acids as contributing factors in carcinogenesis.. J Natl Cancer Inst.

[OCR_01711] Singer B. (1977). Sites in nucleic acids reacting with alkylating agents of differing carcinogenicity of mutagenicity.. J Toxicol Environ Health.

[OCR_01729] Sista H. S., Loder R. T., Caruthers M. H. (1979). Studies on gene control regions X. The effect of specific adenine-thymine transversions on the lac repressor-lac operator interaction.. Nucleic Acids Res.

[OCR_01745] Smith R. C., Salmon W. D. (1965). Formation of S-adenosylethionine by ethionine-treated rats.. Arch Biochem Biophys.

[OCR_01764] Stoner G. D., Shimkin M. B., Kniazeff A. J., Weisburger J. H., Weisburger E. K., Gori G. B. (1973). Test for carcinogenicity of food additives and chemotherapeutic agents by the pulmonary tumor response in strain A mice.. Cancer Res.

[OCR_01771] Sutter D., Doerfler W. (1980). Methylation of integrated adenovirus type 12 DNA sequences in transformed cells is inversely correlated with viral gene expression.. Proc Natl Acad Sci U S A.

[OCR_01777] Swann P. F., Pegg A. E., Hawks A., Farber E., Magee P. N. (1971). Evidence for ethylation of rat liver deoxyribonucleic acid after administration of ethionine.. Biochem J.

[OCR_01783] Takebe H., Miki Y., Kozuka T., Furuyama J. I., Tanaka K. (1977). DNA repair characteristics and skin cancers of xeroderma pigmentosum patients in Japan.. Cancer Res.

[OCR_01795] Vogler W. R., Miller D. S., Keller J. W. (1976). 5-Azacytidine (NSC 102816): a new drug for the treatment of myeloblastic leukemia.. Blood.

[OCR_01800] Von Hoff D. D., Slavik M., Muggia F. M. (1976). 5-Azacytidine. A new anticancer drug with effectiveness in acute myelogenous leukemia.. Ann Intern Med.

[OCR_01806] Waechter D. E., Baserga R. (1982). Effect of methylation on expression of microinjected genes.. Proc Natl Acad Sci U S A.

[OCR_01811] Waldstein E. A., Cao E. H., Setlow R. B. (1982). Adaptive resynthesis of O6-methylguanine-accepting protein can explain the differences between mammalian cells proficient and deficient in methyl excision repair.. Proc Natl Acad Sci U S A.

[OCR_01818] Weinberg A. G., Mize C. E., Worthen H. G. (1976). The occurrence of hepatoma in the chronic form of hereditary tyrosinemia.. J Pediatr.

[OCR_01823] Weinberg R. A. (1982). Oncogenes of spontaneous and chemically induced tumors.. Adv Cancer Res.

[OCR_01845] Weinhouse S. (1980). New dimensions in the biology of cancer.. Cancer.

[OCR_01849] Yuan R., Meselson M. (1970). A specific complex between a restriction endonuclease and its DNA substrate.. Proc Natl Acad Sci U S A.

[OCR_01225] de Wachter R., Merregaert J., Vandenberghe A., Contreras R., Fiers W. (1971). Studies on the bacteriophage MS2. The untranslated 5'-terminal nucleotide sequence preceding the first cistron.. Eur J Biochem.

